# Effects of high temperature on photosynthesis and related gene expression in poplar

**DOI:** 10.1186/1471-2229-14-111

**Published:** 2014-04-28

**Authors:** Yuepeng Song, Qingqing Chen, Dong Ci, Xinning Shao, Deqiang Zhang

**Affiliations:** 1 National Engineering Laboratory for Tree Breeding, College of Biological Sciences and Technology, Beijing Forestry University, No. 35, Qinghua East Road, Beijing 100083, P. R. China; 2 Key Laboratory of Genetics and Breeding in Forest Trees and Ornamental Plants, College of Biological Sciences and Technology, Beijing Forestry University, No. 35, Qinghua East Road, Beijing 100083, P. R. China

**Keywords:** Photosynthesis, Gene expression profile, Heat stress, *Populus simonii*

## Abstract

**Background:**

High temperature, whether transitory or constant, causes physiological, biochemical and molecular changes that adversely affect tree growth and productivity by reducing photosynthesis. To elucidate the photosynthetic adaption response and examine the recovery capacity of trees under heat stress, we measured gas exchange, chlorophyll fluorescence, electron transport, water use efficiency, and reactive oxygen-producing enzyme activities in heat-stressed plants.

**Results:**

We found that photosynthesis could completely recover after less than six hours of high temperature treatment, which might be a turning point in the photosynthetic response to heat stress. Genome-wide gene expression analysis at six hours of heat stress identified 29,896 differentially expressed genes (15,670 up-regulated and 14,226 down-regulated), including multiple classes of transcription factors. These interact with each other and regulate the expression of photosynthesis-related genes in response to heat stress, controlling carbon fixation and changes in stomatal conductance. Heat stress of more than twelve hours caused reduced electron transport, damaged photosystems, activated the glycolate pathway and caused H_2_O_2_ production; as a result, photosynthetic capacity did not recover completely.

**Conclusions:**

This study provides a systematic physiological and global gene expression profile of the poplar photosynthetic response to heat stress and identifies the main limitations and threshold of photosynthesis under heat stress. It will expand our understanding of plant thermostability and provides a robust dataset for future studies.

## Background

Photosynthesis converts light energy into usable chemical energy for plant growth and development [1]. As the most intricate physiological process in plants, photosynthesis incorporates numerous components, including CO_2_ reduction pathways, photosynthetic photosystems and the electron transport system [2]. Among these, Photosystem II (PSII) has been described as the most heat-sensitive component of the photosynthetic apparatus [3]. In *Populus euphratica*, heat stress causes a decrease in PSII abundance and an increase of Photosystem I (PSI); it also induces photosynthetic linear electron flow [4]. Sharkey et al. (2005) reported that reduction of plastoquinone and cyclic electron flow can be stimulated by moderate heat stress [5]. Moderate heat stress also causes a reduction in Rubisco activities. The Rubisco oxygenase side reaction promotes the production of H_2_O_2_, which can be toxic to plant cells.

Transitory or constant high temperature causes morphological, physiological, and biochemical changes that reduce photosynthesis and thus limit plant growth and productivity [2,6]. Moderate heat stress causes a reversible reduction of photosynthesis; increased heat stress causes irreversible damage to the photosynthetic apparatus, resulting in greater inhibition of plant growth [7]. A report from the Intergovernmental Panel on Climatic Change [8], predicts that the Earth’s climate will warm by 2–4°C by the end of the 21st Century [9]. Therefore, a fundamental understanding of the response of photosynthetic physiology and related gene expression under heat stress may help to improve the thermostability of plants and limit the adverse effects of climate change on crop yield.

Many studies have examined the effects of stress on the electron transport system, photosystems, pigments, photosynthesis-related enzyme activities, gas exchange and chlorophyll fluorescence in plants [10,11]. These studies have mostly focused on the adaptive responses of plants to heat stress, but less attention has been paid to the recovery capacity of plants under stress. Trees, with their long lifetimes, must periodically contend with fluctuating environmental conditions. Thus, they have evolved specific physiological mechanisms to adapt to natural changes in environmental conditions [12]. Analysis of the adaption response and recovery capacity of trees to heat stress will expand our understanding of thermostability in all plants.

Most adaptive responses function, at least in part, through control of gene expression; therefore, heat-responsive transcription factors might play a critical role in abiotic stress responses [13]. Multiple genes interacting with each other and with the environment act in the responses to heat stress [2]. bZIP (Basic Leucine Zipper) transcription factors have broad functions in plant biotic and abiotic stress responses, light signaling, and abscisic acid (ABA) signaling [14]. NAC (NAM, ATAF1/2, CUC2) family transcription factors have been implicated in the activation of expression of *EARLY RESPONSIVE TO DEHYDRATION STRESS 1* (*ERD1*) [15] and are predominantly induced by abiotic stress in guard cells [16]. *MYB* gene family members function in ABA signaling, and in jasmonic acid-related gene expression, indicating that they affect crosstalk between abiotic and biotic stress responses [17]. CBF/DREB1 (C Repeat Binding Factor/Drought Response Element Binding 1) family members activate the expression of genes related to the production of osmoprotectants and antioxidants and their expression is quickly and transiently induced by abiotic stress [13]. The numbers and expression of genes involved in regulation of photosynthesis in trees in response to heat stress remains unclear. Therefore, it is extremely important to identify and analyze genes involved in high temperature tolerance in trees.

The advantages of using members of the poplar genus (*Populus*) as genomic models for tree molecular biology have been extensively reported [18,19]. Among *Populus* species, *P. simonii* shows remarkable survival capability, even in extreme temperatures (-41°C to +43°C) and other abiotic stresses [20]. Recent work reported the genome-wide gene expression profiles of the *P. simonii* responses to chilling and drought stress [21,22]. However, information on the genome-wide transcriptome response of *P. simonii* to heat stress remains limited. Therefore, we selected *P. simonii* to examine the mechanisms of heat-tolerance in poplar. Our study presents a systematic investigation of differentially expressed genes in heat-stressed *P. simonii*. Furthermore, these differentially expressed genes may be suitable targets for biotechnological manipulation to improve heat tolerance in poplar and other species.

## Methods

### Plant materials and treatments

*P. simonii* samples were collected from Huzhu County of Qinghai Province, northwest China. The 1-year-old plant material was propagated from branches of adult mother plants. The material was planted in pots with inner size of 10 cm in height and 15 cm in diameter, containing a potting mix of a commercial medium and perlite at a ratio of 3:1. These seedlings were watered regularly with a nutrient solution.

Poplar ‘QL9’ were maintained under natural light conditions in an air-conditioned greenhouse under a 25 ± 1°C, 50% ± 1 relative humidity and 12 h day/ night regime [23]. Fifty clones were propagated from mother plant ‘QL9’. Among these, fifteen annual clones of approximately the same size and height were exposed to constant high temperature treatment (42°C) for three hours, six hours, twelve hours and twenty-four hours. Clones growing at constant room temperature (25°C) were used as the control group. Relative humidity set to 50% ± 1 was held constant during measurements [24]. Each treatment group, including the control group, contained three replicate clones. Gas exchange and chlorophyll a fluorescence transients were measured under stress conditions. To detect the recovery of photosynthesis under heat stress, each treatment group was returned to room temperature after 24 h, then gas exchange and chlorophyll a fluorescence transients were measured again. To confirm whether candidate genes were generally temperature-responsive, constant chilling stress (4°C, six hours) were performed. Constant 1250 μmolm^-2^s^-1^ PPFD light conditions were provided during treatment. Leaves were collected from treatment groups and the control group for physiological and gene expression analysis, then immediately frozen in liquid nitrogen and stored at -80°C until analyzed.

### Photosynthetic rate measurements

The fourth fully expanded leaf, from each of three clones in each treatment was harvested for photosynthetic rate measurements using the portable photosynthesis system (LI-6400; Li-Cor Inc., Lincoln, NE, USA) from 18 to 24 August 2013. To achieve full photosynthetic induction, all samples were illuminated with saturated photosynthetic photon flux density (PPFD) provided by a light-emitting diode (LED) light source for 30 min before measurements. Subsequently, net photosynthetic rate (Pn), transpiration rate (Tr), intercellular CO_2_ concentration (Ci) and stomatal conductance (Gs) were measured simultaneously. All parameters for measurement were as described by Chen et al. (2010) [25]. Intrinsic water use efficiency (iWUE) was calculated from the ratio of Pn and Tr.

### Measurement of physiological and biochemical characteristics

Superoxide dismutase (SOD), peroxidase (POD), catalase (CAT) and malondialdehyde (MDA) were measured as described by Giannopolitis and Ries (1977), Bestwick et al*.* (1998), Carrill et al. (1992) and Dhindsa et al. (1981), respectively, and measured by absorption photometry using a spectrophotometer. The details were according to Song et al. (2013) [26-30]. Ascorbate peroxidase (APX) activity assays were according to the method of Nakano and Asada (1981) [31]. At 290 nm, absorbance of the reaction was monitored using a spectrophotometer. The extinction coefficient of ascorbate was used for calculating APX enzyme activity.

### H_2_O_2_ analysis

Endogenous H_2_O_2_ levels were detected by measuring luminol-dependent chemiluminescence according to the method described by Dat et al. (1998) and the H_2_O_2_-specific fluorescent probe 2',7'-Dichlorodihydrofluorescein diacetate (H2DCF-DA, green) (Molecular Probes, Eugene, OR, USA, prepared in a 2-(N-morpholino) ethane sulfonic acid (MES)-KCl buffer, pH 5.7) [32]. MES-KCl buffer solution was used for washing the leaves sampled from treated poplar. After that, all samples were incubated in the buffer solution containing 50 μM H2DCF-DA for 40 min at room temperature. Leaves were examined using a Leica SP5 confocal microscope (Leica Microsystems GmbH, Wetzlar, Germany) under the following settings: excitation = 488 nm, emission = 510–530 nm, frame 512 × 512.

### Chlorophyll fluorescence measurement

Chlorophyll fluorescence was measured using the LICOR 6400 system, according to the recommended procedures in the users’ manual (LICOR Biosciences, Inc., Lincoln, NE). The fourth fully expanded leaves were dark-acclimated in the LI-6400XT leaf chamber for 20 min at 28 ± 0.1°C prior to measuring minimum fluorescence (Fo) and maximum fluorescence (Fm), which was followed by 20 min of light acclimation at 550 μmol m^-2^s^-1^ PPFD prior to ramping up temperature [33]. Variable fluorescence (Fv) in the dark-adapted state was calculated as: Fv = Fm-Fo. The fluorescence chamber provided a one-second pulse of continuous red light (3000 μmol photons m^-2^s^-1^ maximum light intensity) for illumination. Maximum quantum efficiency of PSII was calculated using the formula: Fv/Fm = (Fm-Fo)/Fm. Subsequently, the minimum fluorescence (F′o), variable fluorescence (F′v) and maximum fluorescence (F′m) in the light-adapted state were measured. Photochemical quenching (qP) was calculated as: qP = (F′m-Fs)/(F′m-F′o) using the steady state parameter (Fs). Simultaneously, the relative quantum yield of PS II (φPSII) was calculated as: φPSII = (F′m-Fs) /F′m and the electron transport rate (ETR) was estimated as: ETR = PPFD × φPSII × 0.85 × 0.5.

### RNA extraction, cDNA synthesis, Microarray Hybridization and Data Analysis

RNAeasy Plant mini kit (Qiagen, Hilden, Germany) and Super-Script First-Strand Synthesis system (Invitrogen) were used for total RNA extraction and cDNA synthesis, respectively. The details were according to the method described by Song et al. (2013) [30]. To identify differentially expressed genes under heat stress, we used the six-hour treatment group for microarray expression profiling. Fresh tissue leaf samples were collected from the three independent *P. simonii*, as biological replicates, for RNA extraction. The process of amplification, labeling, purification and hybridization were performed at the Shanghai Bio Institute using the Affymetrix GeneChip Poplar Genome Array (contained 6, 1314 probe). Gene set enrichment analysis was performed using AgriGO analysis tools (http://bioinfo.cau.edu.cn/agriGO/). Annotation information was obtained from GenBank (http://www.ncbi.nlm.nih.gov/genbank/) and The Kyoto Encyclopedia of Genes and Genomes (KEGG, http://www.genome.jp/kegg).

### Quantitative Real-time Polymerase Chain Reaction (PCR) verification

Quantitative PCR (qPCR) was performed using the TaKaRa ExTaq R PCR Kit, SYBR green dye (TaKaRa, Dalian, China) and a DNA Engine Opticon 2 machine (MJ Research). The qPCR program included an initial denaturation at 94°C for 5 min, followed by 40 cycles of 30 s at 94°C, 30 s at 58°C, and 30 s at 72°C, and a final melt-curve 70–95°C. The melting curve was used to check the specificity of the amplified fragment. All reactions were carried out in triplicate for technical and biological repetitions of three individuals. The generated real-time data were analyzed using the Opticon Monitor Analysis Software 3.1 tool. Specific primer sets were designed to target the 3′ untranslated region (UTR) of each gene using Primer Express 3.0 software (Applied Biosystems). The real-time PCR primer pairs are shown in Additional file 1. The efficiency of the primer sets was calculated by performing real-time PCR on several dilutions of first-strand cDNAs. Efficiencies of the different primer sets were similar. The specificity of each primer set was checked by sequencing PCR products [34]. The results obtained for the different tissues analyzed were standardized to the transcript levels for *PtACTIN* (Additional file 2).

### Statistical analysis

One-way ANOVA was performed using the R software, and significant differences between different stress treatments were determined through Fisher's Least Significant Difference (LSD) test. Differences were considered statistically significant when P < 0.01. Differentially expressed genes (fold change >2 or <0.5; *P* < 0.001) were identified. The parameters of fold change analysis data filtered and minimum false discovery rate were calculated according to Song et al. (2013) [21].

## Results

### Response of the photosynthetic rate to heat stress

**Figure 1:**
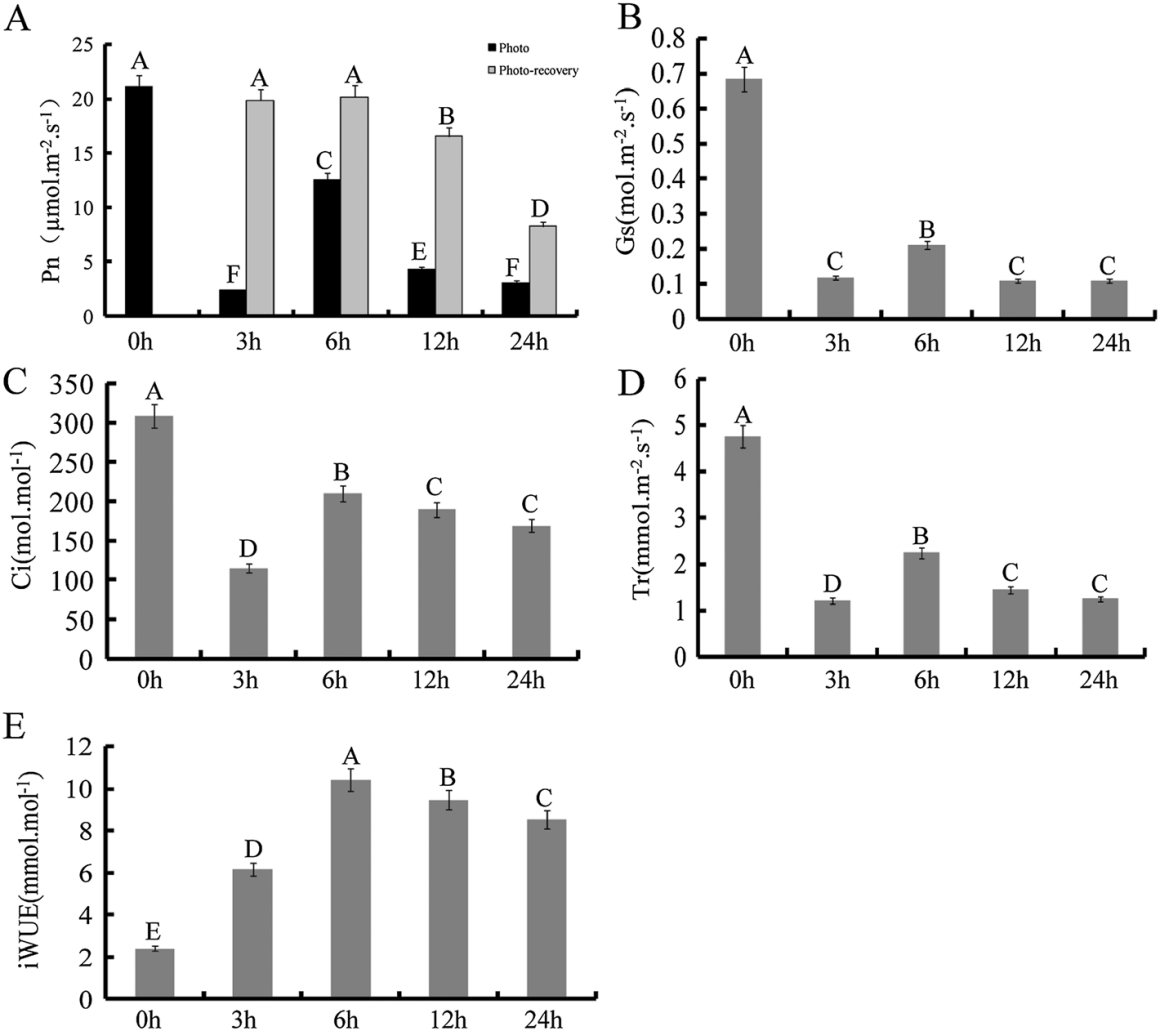
**Changes in gas exchange at high temperatures. A**: Pn represents photosynthetic rate; **B**: Gs represents stomatal conductance; **C**: Ci represents intercellular CO_2_ concentration; **D**: Tr represents transpiration rate; **E**: iWUE represents intrinsic water use efficiency. 0h indicates the control group without high temperature treatment. 3-24h indicates different times of exposure to heat stress. Error bars represent standard error. Different letters on error bars indicate significant differences at P < 0.01. Symbols are the same in the following figures.

To examine the effects of high temperature on poplar photosynthesis, we measured the dynamic Pn, Ci, Gs, Tr, and iWUE over a time course of high temperature treatment (0 h, 3 h, 6 h, 12 h, 24 h) (Figure 1A-E). At three hours, Pn, Gs, Tr and Ci were significantly lower in heat-treated plants than in control plants, but iWUE was significantly higher. At six hours, Pn, Gs and Tr increased slightly in heat-treated plants, but were significantly less than in control plants. Also at six hours, Ci decreased to its minimum value and iWUE increased dramatically to a peak. After six hours, Pn, Gs, Tr and iWUE decreased from twelve to twenty-four hours. By contrast, Gi showed a rising trend at subsequent time points.

**Figure 2:**
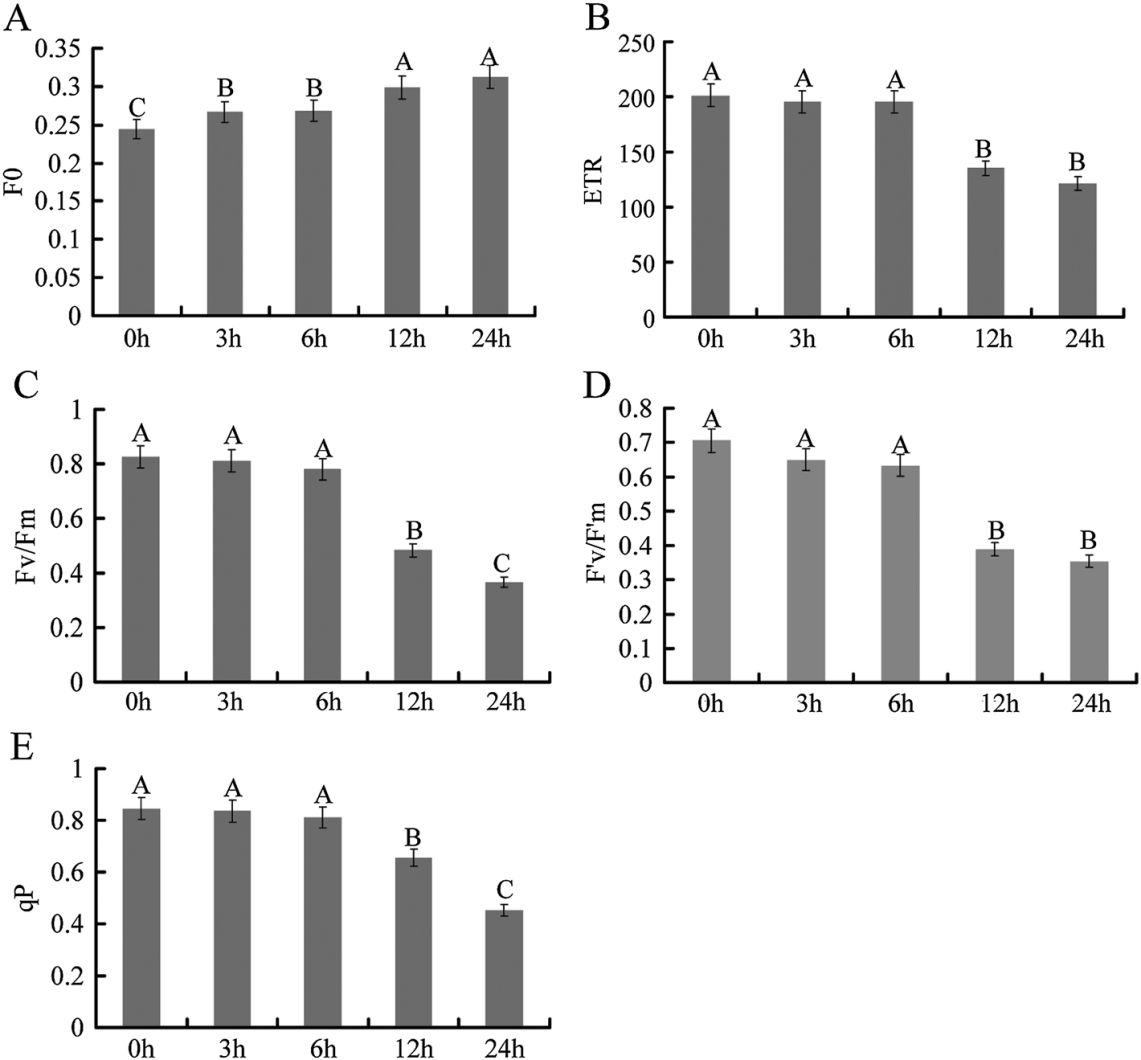
**Changes in chlorophyll fluorescence at high temperatures. A**: Fo represents minimum fluorescence. **B**: ETR represents electron transport rate. **C**: Fv/Fm represents the ratio of variable to maximal chlorophyll fluorescence. **D**: F′v/F′m represents fluorescence in the light ratio. **E**: qP represents photochemical quenching. 0 h indicates the control group without high temperature treatment. 3-24 h indicates different times of exposure to heat stress. Error bars represent standard error. Different letters on error bars indicate significant differences at P < 0.01.

We also detected photosynthetic recovery after heat stress at different time points in plants that had been returned to room temperatures. Our results showed that photosynthetic rate could be completely recovered after three or six hours of high temperature treatment. However, after 12 h and 24 h heat stress, the photosynthetic rate recovered to only 68.8% and 45.2% of control group levels, respectively. Chlorophyll fluorescence reflects the photodamage or photoprotection-related effects of environmental stress on photosynthetic systems [35]. To examine this, we measured Fo, the ratio of variable and maximal fluorescence (Fv/Fm), electron transport rate (ETR) and fluorescence quenching coefficient (*q*P) (Figure 2A-E). Compared with the control group, Fo, Fv/Fm, F′v/F′m ETR and *q*P were not significantly changed after three and six hours heat stress. After that, Fv/Fm, F′v/F′m, ETR and *q*P dramatically decreased at 12 and 24 h, but Fo increased significantly and constantly.

### Changes in physiological and biochemical parameters in response to heat stress

**Figure 3:**
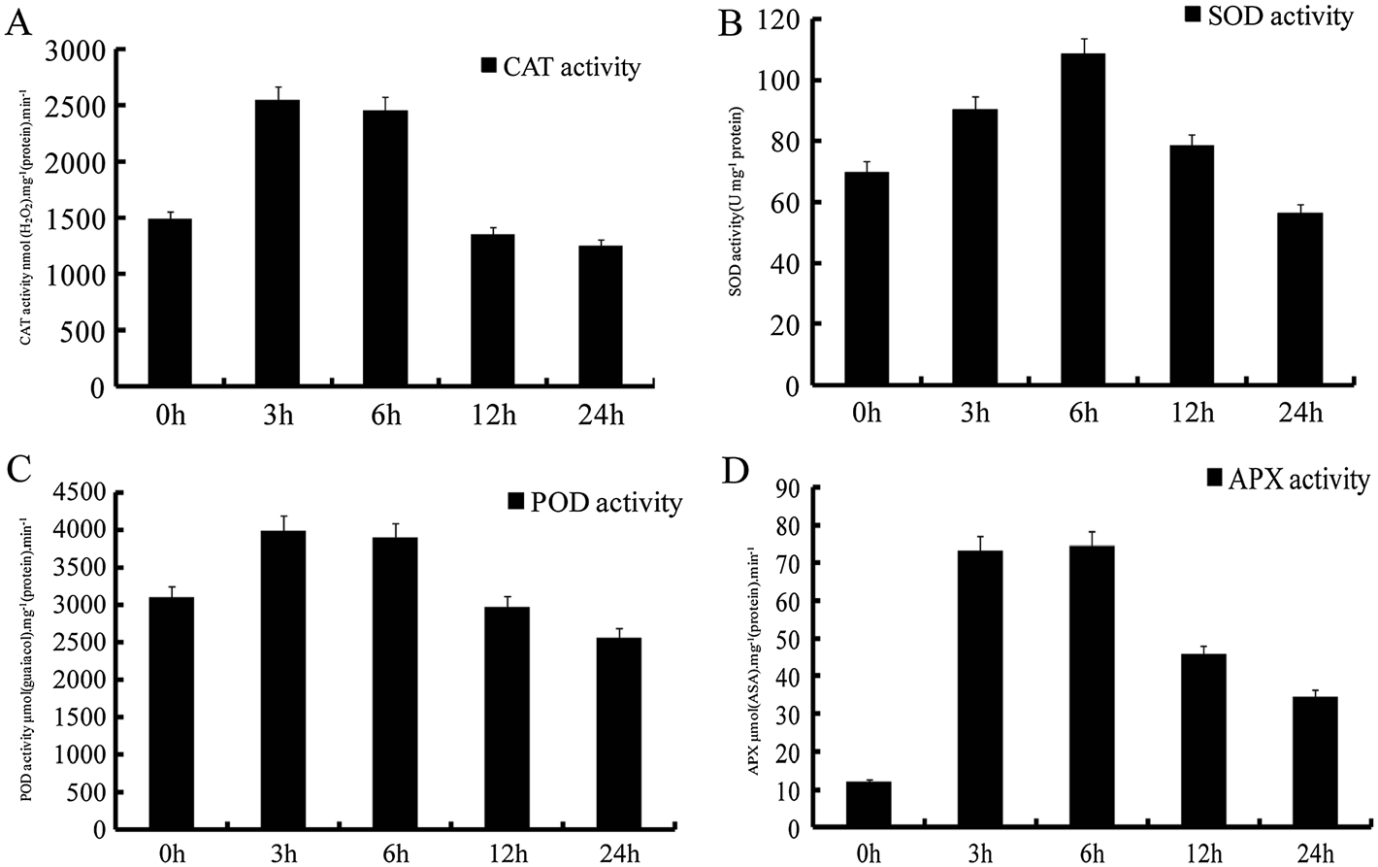
**Change of SOD, POD CAT and APX activities response to heat stress. A**: SOD activities; **B**: POD activities; **C**: CAT activities; **D**: APX activities. Activities are presented as means ± standard error, and n = 3.

Antioxidant enzymes buffer oxidative stress caused by high temperature. Therefore, we measured the activities of four antioxidant enzymes, SOD, POD CAT and APX (Figure 3). High temperature significantly increased the activities of all antioxidant enzymes at three hours. Subsequently, POD, CAT and APX activities showed no significant change at six hours, but SOD activity sharply increased during exposure to high temperature. After six hours, all four antioxidant enzyme activities decreased from 12 h to 24 h.

**Figure 4:**
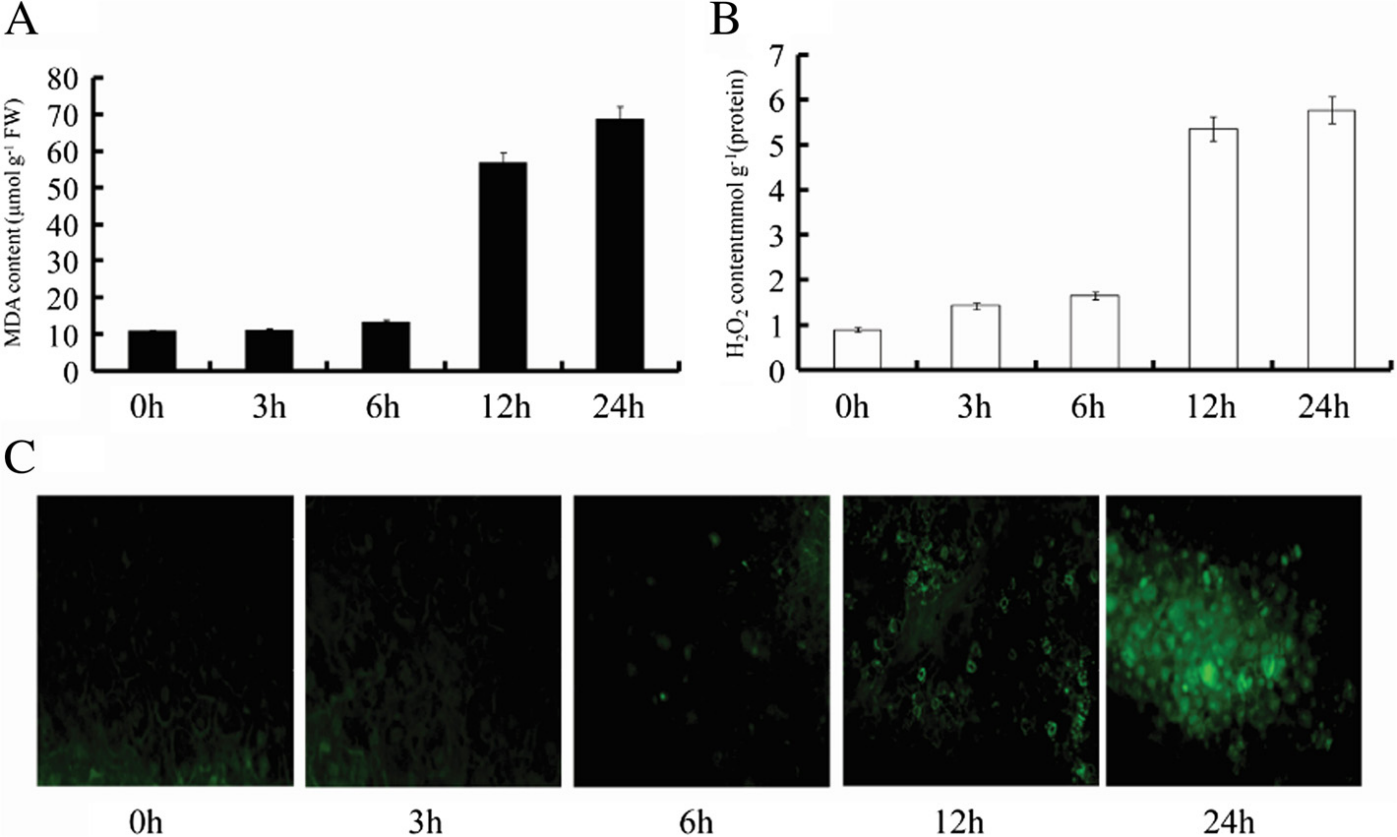
**Concentration of MDA and H**_**2**_**O**_**2 **_**in leaves of poplar under high temperature. A**: concentration of MDA under heat stress. **B**: concentration of H_2_O_2_ under heat stress. **C**: the changes of H_2_O_2_ under heat stress were detected by H_2_O_2_-specific fluorescent probe H2DCF-DA (green). 0h indicates control group without high temperature treatment. 3-24h indicates different times of exposure to heat stress. Concentrations are presented as means ± standard error, and n = 3.

If cellular antioxidants do not sufficiently counter the oxidative stress induced by heat stress, cellular reactive oxygen may cause lipid peroxidation. Therefore, we measured MDA content, a classic marker of lipid peroxidation. The MDA concentration of poplar did not change under heat stress at three hours and six hours of stress treatment but increased and peaked at the 24 h time point (Figure 4A). To measure endogenous H_2_O_2_ levels, we used an H_2_O_2_-specific fluorescent probe and spectrophotometry. H_2_O_2_ production slightly changed after six hours heat stress and then increased by 3.4-fold, at 12 h and 24 h (Figure 4B and C).

### A portrait of the poplar transcriptional response to heat stress

Measurement of photosynthetic physiological characteristics showed that Pn and iWUE increased significantly after high temperature treatment for six hours, implying there might be a substantial change in gene expression at this time point (Figure 1A and E). Identifying differentially expressed genes might provide new insights into how poplar maintains photosynthesis under heat stress. Therefore, we used the six hour high temperature treatment group for microarray expression profiling.

Microarray analysis identified 29,896 reliable signatures that were differentially expressed (Fold change >2 or <0.5; P < 0.001) between treatments and controls; of these, 15,670 were up-regulated and l4,226 were down-regulated. Comparative analyses indicated that the highest and lowest expression ratios (heat treated/control) were 2677 and 0.0097, respectively.

**Figure 5:**
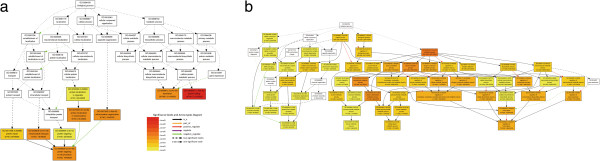
**Differentially expressed genes in response to heat stress for statistically enriched GO terms in the “Biological process” ontology.***P*-values < 0.05 are shown in parentheses. Coloring of GO term nodes is proportional to their significance, as indicated by the scale. **A**. AgriGO analysis of genes up-regulated under heat stress. **B**. AgriGO analysis of genes down-regulated under heat stress.

Gene ontology (GO) supplies a unified and structured classification, to specifically describe genes and their products and allows comparison of results from different species. To explore the biological functions of heat-responsive genes, we identified 1,805 genes showing significant differential expression at *P*  <  0.001 and with expression ratios greater than four-fold as candidate genes for functional enrichment analysis. We then characterized these genes functionally using GO terms (Figure 5); this revealed that eight GO terms for biological process were enriched, including protein folding, mitochondrial transport, protein localization in mitochondrion, protein targeting to mitochondrion, translation, mitochondrion organization, protein import, and protein targeting (Figure 5A). For cellular component, GO analysis revealed that the categories cytoplasm, intracellular part, intracellular, intracellular organelle and organelle were enriched. For categories based on molecular function, the genes were classified into 16 categories. The two most overrepresented GO terms were structural constituent of ribosome and structural molecule activity (Additional file 3).

For the heat-repressed genes, differentially expressed genes were related to 11 biological processes including metabolic process, primary metabolic process, cellular metabolic process, nitrogen compound metabolic process, biosynthetic process, cellular process, cellular macromolecule metabolic process and macromolecule metabolic process (Figure 5B). For cellular component, the set of GO terms enriched for the heat-repressed genes was similar to those enriched for heat-induced genes. For molecular functions, the down-regulated genes were classified into six categories including catalytic activity, hydrolase activity, cation binding, metal ion binding, ion binding, and zinc ion binding (Additional file 4).

### Response of expression of photosynthesis-related genes to heat stress

**Figure 6:**
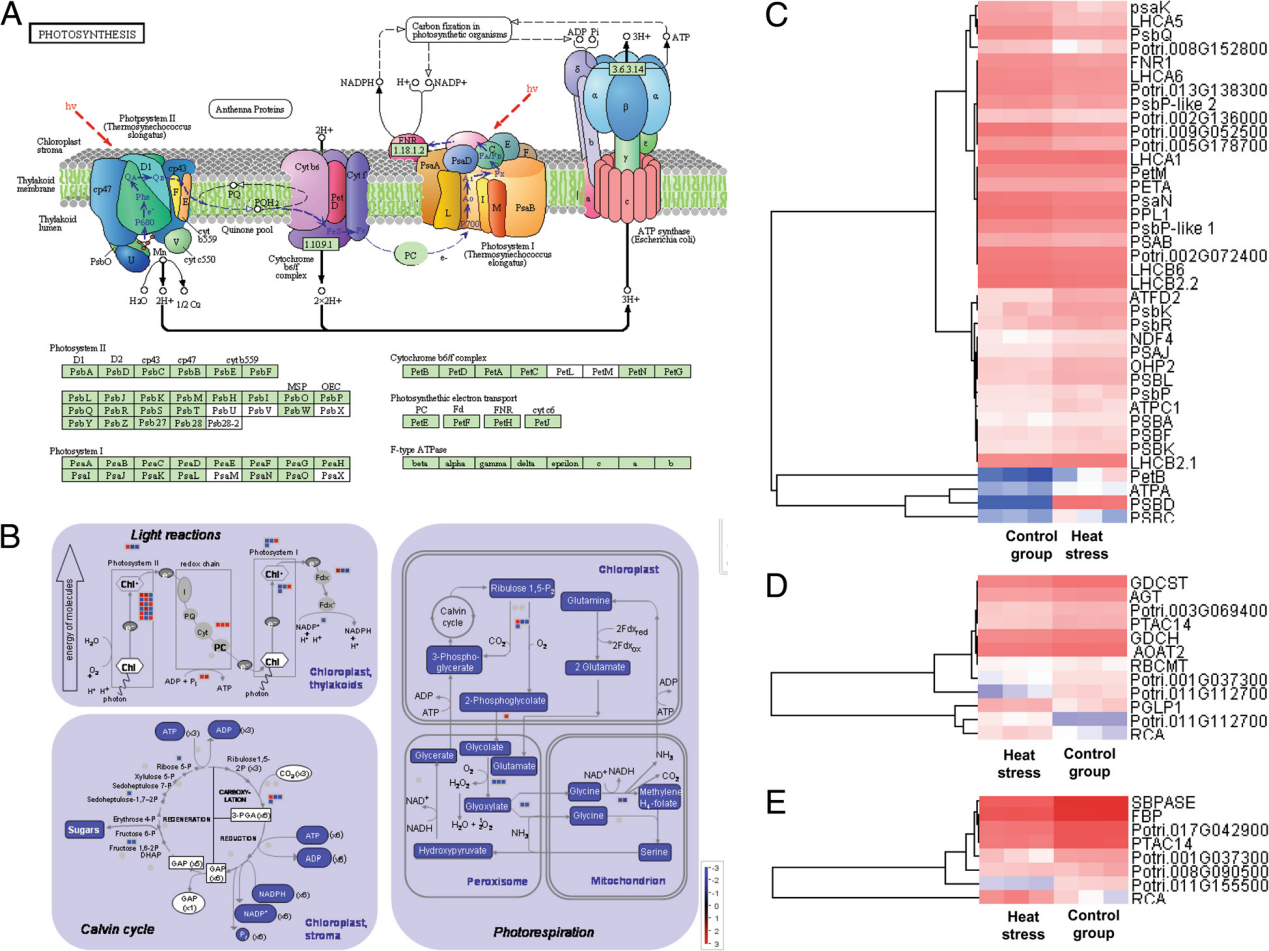
**Diagram of differentially expressed genes involved in photosynthesis. A**: photosynthesis pathway (reference KEGG) **B**: The ‘photosynthesis’ MapMan pathway was used to visualize transcriptional changes in genes with putative functions in metabolism. Red represents higher expression in heat stress samples and blue denotes higher expression in controls, with darker shading indicating increasing magnitude of log2 expression fold change, as specified by the scale. **C**: Pearson correlation coefficient heat map indicating the differentially expressed genes related to photosynthesis. **D**: Pearson correlation coefficient heat map indicating the differentially expressed genes related to photorespiration. **E**: Pearson correlation coefficient heat map indicating the differentially expressed genes related to calvin cycle. Red and blue indicate higher and lower transcript levels, respectively. The gene model is shown on the right. The control group consisted of three biological samples that were not treated with high temperature.

Base on the MapMan analysis, fifty-six photosynthesis-related genes were detected as differentially expressed in the response to heat stress. Among these, twenty-one genes were up-regulated, including eighteen genes involved in light reactions, one gene in the Calvin cycle and two genes for photorespiration (Table 1 and Figure 6). Thirty-six photosynthesis-related genes were repressed under heat stress. Among these, twenty, seven and nine genes are involved in the light reaction, Calvin cycle and photorespiration, respectively.

**Table 1:** Upregulated-genes involved in photosynthesis in the response to heat stress

**Biological process**	**Location**		**Alias**	**Gene model**	**Description**	**TAIR gene model**	**Fold change**
Light reaction	Chloroplast thylakoids	PS I	LHCB2.1	Potri.014G165100	chlorophyll a/b-binding protein	AT2G05100	2.61
			PSBF	Potri.011G095300	PSII cytochrome b559	ATcG00570	2.21
			PSBK	Potri.013G138100	PSII PsbK protein	ATcG00070	2.02
			PSBC	Potri.008G207300	PSII protein	ATcG00280	6.12
				Potri.002G072400	thylakoid lumenal 29.8 kDa protein	AT1G77090	2.37
			PSBL	Potri.002G237300	PSII L protein	ATcG00560	3.97
			PSBA	Potri.013G138300	Photosynthetic reaction centre protein	ATcG00020	2.02
			PSBK	Potri.013G138100	PSII PsbK protein	ATcG00080	4.84
			PSBD	Potri.008G207200	PSBD | PSII D2 protein	ATcG00270	183.90
			PSBR	Potri.011G142200	PSII 10 kDa polypeptide	AT1G79040	3.28
		Redox chain	PETA	Potri.T058600	electron carrier activity	ATcG00540	9.14
			PETM	Potri.004G003000	cytochrome b6f complex subunit (petM)	AT2G26500	9.18
			PETB	Potri.013G137300	Cytochrome b(N-terminal)/b6/petB	ATcG00720	24.80
			ATPA	Potri.013G138000	ATP synthase alpha/beta family,	ATcG00120	5.01
		PS II	ATPC1	Potri.004G014800	ATP synthase gamma chain 1	AT4G04640	2.38
			OHP2	Potri.005G196100	a novel member of the Lhc family	AT1G34000	3.54
			PSAJ	Potri.003G067400	Encodes subunit J of PS I	ATcG00630	2.74
			NDF4	Potri.001G186800	Ribosomal protein L33	AT3G16250	2.09
Calvin cycle	Chloroplast stroma		RCA	Potri.008G058500	Ribulose bisphosphate carboxylase/oxygenase activase	AT2G39730	6.21
Photo-respiration	chloroplast		RCA	Potri.008G058500	Ribulose bisphosphate carboxylase/oxygenase activase	AT2G39730	6.21
			PGLP1	Potri.008G077400	2-phosphoglycolate phosphatase 1	AT5G36700	4.57

In the light reaction, fourteen differentially expressed genes affected PSI, including ten up-regulated genes and four down-regulated genes. In contrast, twenty differentially expressed genes were detected for PSII, four up-regulated genes and sixteen down-regulated genes. The observation that more genes were down-regulated than up-regulated suggested that PSII might suffer more negative effects from heat stress than PSI (Figure 6). Also, all four genes for the redox chain (*PETA*, *PETM*, *PETB* and *ATPA*) were significantly up-regulated after six hours heat stress, suggesting these genes might play important roles in maintaining electron transfer in photosynthesis under heat stress (Table 2).

**Table 2:** Downregulated-genes involved in photosynthesis in the response to heat stress

**Biological process**	**Location**		**Alias**	**Gene model**	**Description**	**TAIR gene model**	**Fold change**
Light reaction	Chloroplast thylakoids	PS I	LHCB2.2	Potri.014G165100	similar to chlorophyll a/b-binding protein - garden pea	AT2G05070	0.48
			LHCB6	Potri.001G210000	similar to chlorophyll A-B binding protein;	AT1G15820	0.44
			PNSL	Potri.010G210000	PSII reaction center PsbP family protein	AT2G39470	0.29
				Potri.008G152800	similar to PSII 11 kDa protein-related	AT1G05385	0.33
		PS II		Potri.005G178700	oxygen-evolving complex-related	AT1G76450	0.23
				Potri.009G052500	similar to PSII 5 kD protein	AT1G51400	0.12
				Potri.007G100800	a PsbP domain-OEC23 like protein localized in thylakoid	AT2G28605	0.07
				Potri.005G206200	PSII family protein	AT1G03600	0.22
			PPL1	Potri.010G210200	PPL1 (PsbP-like protein 1)	AT3G55330	0.23
			PSBQ	Potri.001G416400	oxygen evolving enhancer 3 (PsbQ) family protein;	AT3G01440	0.11
			PSBO1	Potri.005G130400	similar to O_2_ evolving complex 33kD protein	AT5G66570	0.35
			LHCA1	Potri.008G041000	similar to LHCI type I (CAB)	AT3G54890	0.24
			LHCA6	Potri.006G139600	similar to LHCI type II	AT1G19150	0.34
			LHCA5	Potri.014G029700	similar to chlorophyll A-B binding protein	AT1G45474	0.14
			PSAN	Potri.007G105900	PSI subunit Psa N	AT5G64040	0.42
			PSAB	Potri.017G052700	PSI psaA/psaB protein	ATcG00340	0.28
			PSAK	Potri.006G254200	similar to PSI reaction center subunit Psa K;	AT1G30380	0.18
				Potri.002G136000	ferredoxin-related	AT1G02180	0.42
			ATFD2	Potri.004G218400	similar to Ferredoxin 2; similar to chloroplast precursor	AT1G60950	0.44
			FNR1	Potri.007G057200	similar to Chain A;	AT5G66190	0.37
Calvin cycle	Chloroplast stroma			Potri.011G155500	similar to ribose 5-phosphate isomerase-related;	AT5G44520	0.25
				Potri.008G090500	similar to Ribulose-1;	AT1G14030	0.43
				Potri.001G037300	ATPase family associated with various cellular activities	AT1G73110	0.38
			PTAC14	Potri.003G155100	plastid transcriptionally active 14	AT4G20130	0.30
				Potri.017G042900	Fructose-1-6-bisphosphatase	AT5G64380	0.33
			FBP	Potri.016G106900	similar to Redox Signaling In The Chloroplast:	AT3G54050	0.20
			SBPASE	Potri.010G193300	similar to Sedoheptulose-1;	AT3G55800	0.13
Photo- respiration	Chloroplast		RBCMT	Potri.008G090500	similar to Ribulose-1;	AT1G14030	0.43
				Potri.001G037300	ATPase family associated with various cellular activities	AT1G73110	0.38
			PTAC14	Potri.003G155100	Rubisco LSMT substrate-binding	AT4G20130	0.30
			HAOX1	Potri.003G069400	similar to (S)-2-hydroxy-acid oxidase;	AT3G14130	0.40
			GOX3	Potri.011G112700	similar to glycolate oxidase	AT4G18360	0.08
			AOAT2	Potri.008G187400	a protein with glyoxylate aminotransferase activity	AT1G70580	0.14
			AGT	Potri.001G253300	similar to aminotransferase 2	AT2G13360	0.07
			GDCST	Potri.011G006800	similar to T-protein of the glycine decarboxylase complex	AT1G11860	0.39
			GDCH	Potri.003G089300	similar to glycine cleavage system protein H precursor	AT1G32470	0.45

Eight genes for the Calvin cycle were differentially expressed under heat stress (Table 2). Among these, only *RCA* (*RIBULOSE BISPHOSPHATE CARBOXYLASE/OXYGENASE ACTIVASE*) was significantly up-regulated (six-fold change); the others were down-regulated, ranging from 0.41- to 0.13-fold change. *SBP* (*SQUAMOSA PROMOTER BINDING PROTEINS*) functions at a branch point in the Calvin cycle and its transcripts showed the most decrease, a 0.13-fold change. In photorespiration, among 11 differentially expressed genes, only *RCA* and *PGLP1* (*PHOSPHOGLYCOLATE PHOSPHATASE 1*) were up-regulated under high temperature treatment (Table 2). The other genes, including *AOAT2* and *GDCST*, associated with transamination and decarboxylation were markedly repressed (Figure 6).

### Time-course analysis of electron transfer and H_2_O_2_ production related gene expression under heat stress

The photosynthetic analysis revealed an obvious decrease in Pn between the six-hour and twelve-hour heat treatment groups, suggesting that six hours might be a turning point in the photosynthetic response to high temperature treatment. Simultaneously, chlorophyll a fluorescence and physiological analysis indicated that electron transfer rate significantly decreased and large amounts of H_2_O_2_ were generated at twelve hours of high temperature treatment, compared with six hours. Based on these results, we concluded that the inhibition of electron transfer and generation of H_2_O_2_ might cause a reduction of photosynthesis under heat stress. Therefore, based on the transcriptome analysis, we chose four genes (*PETA*, *PETM*, *PETB* and *ATPA*) associated with electronic transfer rate and four genes (*PGLP1*, *GOX1*, *GOX2* and *GOX3*) associated with H_2_O_2_ production as candidate genes for time-course gene expression analysis (Table 3).

**Table 3:** Annotation of 14 candidate genes in the response to heat stress

**NO**	**Alias**^**a**^	**Gene model**	**Putative function**^**b**^	**p-value**^**c**^	**q-value**^**d**^	**Fold change**
1	HSFA6B	Potri.005G214800	Member of Heat Stress Transcription Factor (Hsf) family	5.78 E-03	2.96 E-04	135.79
2	DREB7	Potri.010G183700	Putative dehydration responsive element binding protein 2H	2.59 E-03	3.11E-04	18.26
3	DREB8	Potri.008G073600	Putative dehydration responsive element binding protein 2H	3.07 E-03	7.25E-04	26.42
4	PETA	Potri.T058600	electron carrier activity	6.73 E-06	3.11E-05	9.14
5	PetM	Potri.004G003000	cytochrome b6f complex subunit (petM)	3.34 E-03	1.95 E-04	9.18
6	PetB	Potri.013G137300	Cytochrome b(N-terminal)/b6/petB	6.53E-08	2.46 E-04	24.80
7	ATPA	Potri.013G138000	ATP synthase alpha/beta family,	3.30E-05	2.93 E-04	5.01
8	PGLP1	Potri.008G077400	2-phosphoglycolate phosphatase 1	6.90 E-04	3.53E-05	4.57
9	GOX1	Potri.001G394400	similar to glycolate oxidase	2.53 E-04	4.15E-05	0.052
10	GOX2	Potri.002G027000	similar to glycolate oxidase	4.01 E-04	2.24E-05	0.08
11	GOX3	Potri.011G112700	similar to glycolate oxidase	5.63 E-07	1.66E-05	0.087
12	Lhcb6	Potri.003G020400	Lhcb6 protein, light harvesting complex of PS II	1.25 E-03	3.63E-04	0.09
13	JAZ6	Potri.003G068900	JAZ6 transcript levels rise in response to a jasmonate stimulus	6.53E-08	2.46 E-04	14.15
14	Hsp81.4	Potri.016G003400	HEAT SHOCK PROTEIN 81.4	6.25 E-03	2.15E-05	40.30

Aliases^a^ and Putative function^b^ were derived from Joint Genome Institute (JGI: http://www.jgi.doe.gov/).

p-value^c^ candidate genes that showed consistent signal on the arrays were identified using a *p*-value of <0.001.

q-value^d^ candidate genes were identified using a *q*-value(minimum value of false discover rate)of <0.001.

**Figure 7:**
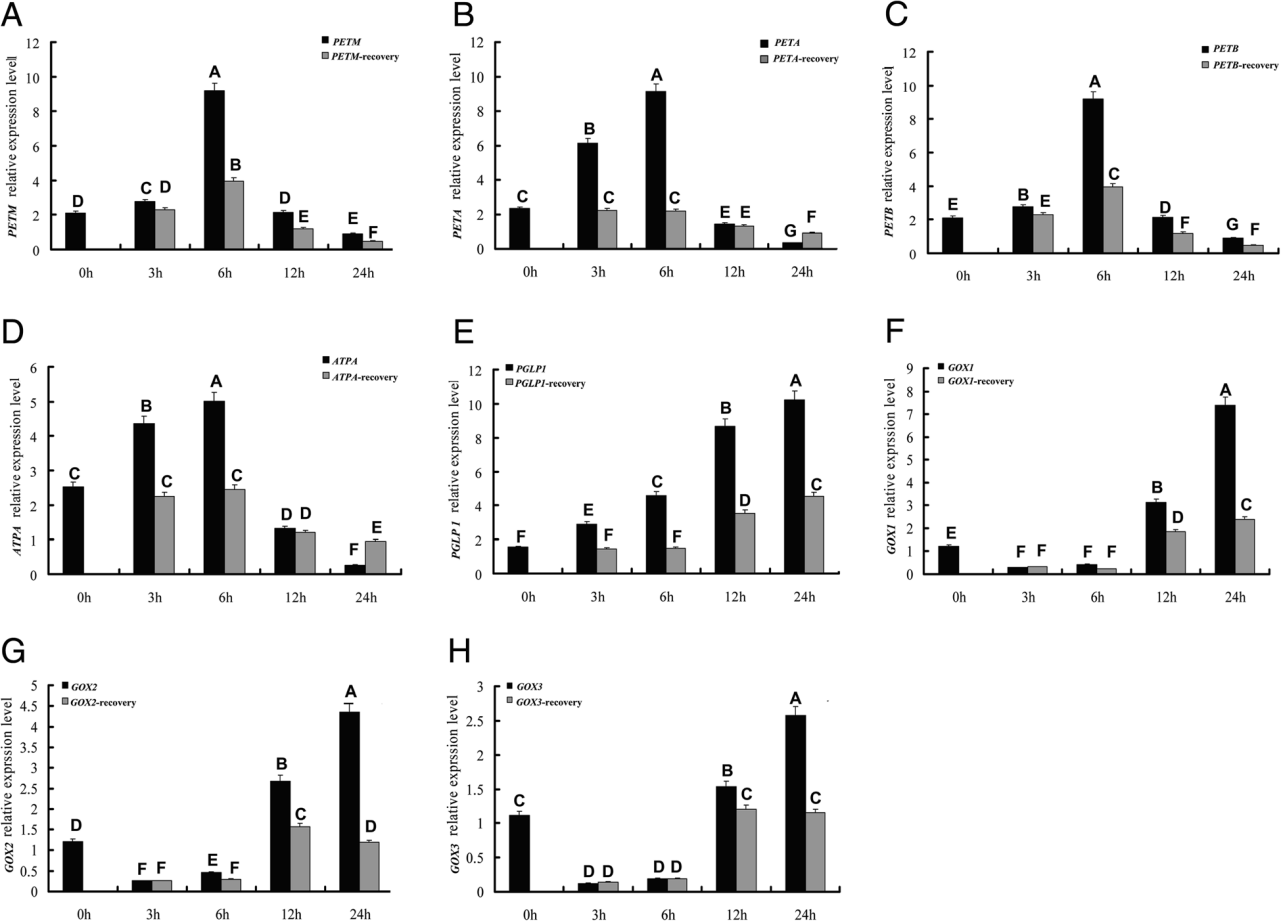
**Quantitative RT-PCR of eight candidate genes related to electron transfers and H**_**2**_**O**_**2 **_**production under heat stress. A-H** represents the expression pattern of *PETM*, *PETA*, *PETB*, *ATPA*, *PGLP1*, *GOX1*, *GOX2* and *GOX3* genes respectively. Transcript levels are normalized to *PtACTIN* and error bars represent standard error. Black column indicates gene expression under heat stress; gray column indicates gene expression after recovery.

All four genes that function to maintain the electronic transfer rate were persistently up-regulated from three hours to six hours, compared with the control group. Subsequently, all of these genes were repressed dramatically at twelve hours and down-regulated to twenty-four hours. After plants were returned to room temperature for twenty-four hours, *PETA* and *ATPA* expression in the three hours and six hours treatment groups recovered to normal levels compared with the control group. In the twelve and twenty-four hour treatment groups, *PETA* and *ATPA* expression recovered to 51% and 74% of control levels, respectively. By contrast, *PETM* and *PETB* expression in the six hours treatment group was higher than in the control group after twenty-four hours of room temperature recovery, suggesting that six hours high temperature treatment could mediate poplar stress adaptation by regulating expression of these two genes (Figure 7).

*PGLP1* plays an important role in the generation of glycolate, which is involved in the glycolate metabolism in photorespiration. *PGLP1* expression increased over time of exposure to high temperature, suggesting that glycolate accumulated constantly. After twenty-four hours at room temperature, *PGLP1* expression completely recovered in the three-hour and six-hour treatment groups. However, *PGLP1* expression of the twelve and twenty-four hour treatment groups was higher than the control group after twenty-four hours of recovery, suggesting that glycollic metabolism was induced by heat stress and might be maintained for a long time.

*GOX* gene family members encode enzymes that catalyze the reaction from glycolate to glyoxylate; this reaction simultaneously produces H_2_O_2_. Three members of the *GOX* gene family were detected in this study and showed two patterns of expression in response to heat stress. At three- and six-hour time points, all three *GOX* genes were down-regulated and completely recovered after treatment ended. *GOX1*, *2* and *3* were significantly induced by heat stress from twelve to twenty-four hours. After recovery, *GOX1* expression was higher than the control group, but *GOX 2* and *3* were not significantly changed compared with the control group. These results suggest that the three *GOX* family members show different expression in response to heat stress.

### Heat regulation of transcription factors related to photosynthesis

**Figure 8:**
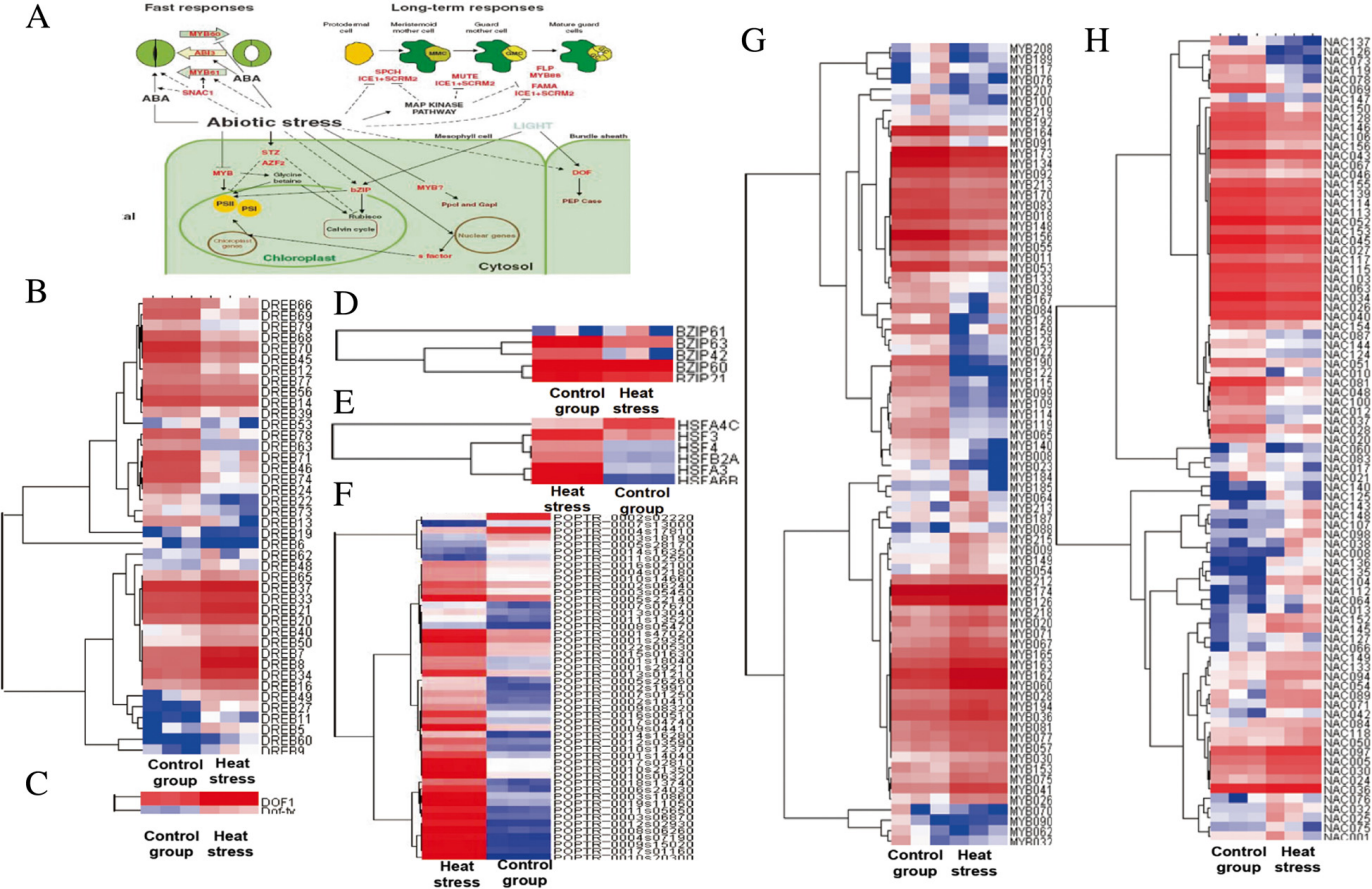
**Expression of candidate transcription factors under heat stress. A**: Diagram of transcription factors involved in stomatal and non-stomatal limitations to CO_2_ photosynthetic assimilation (reference Saibo et al. [13]). Lines with arrowheads represent a positive effect while lines ending with a bar indicate a negative effect. Block arrows show the direction of stomatal movement mediated by the transcription factor. Dashed lines represent possible interactions. **B**: Pearson correlation coefficient (PCC) heat map of *DREB* genes; **C**: PCC heat map of *DOF* genes; **D**: PCC heat map of *bZIP* genes; **E**: PCC heat map of *HSF* genes; **F**: PCC heat map of HSP genes; **G**: PCC heat map of *MYB* genes; **H**: PCC heat map of *NAC* genes.

Transcription factors (TFs) regulate plant abiotic stress responses and mediate stress tolerance [13]. However, only a few TFs are known to regulate the expression of photosynthesis-related genes in response to stress. To understand the expression patterns of TFs that regulate the expression of photosynthesis-related genes under heat stress, we surveyed the expression levels of all TFs using microarray technology. In our study, 165 TF genes were differentially expressed in response to high temperature (Figure 8 and Additional file 5). Among the differentially expressed TF genes, 49 (29.7%) were up-regulated, and 116 (70.3%) were down-regulated.

bZIP transcription factors have broad functions in plant biotic and abiotic stress responses, light signaling, and ABA signaling [14]. Among the sixteen bZIP transcription factors, five bZIPs were differentially expressed under heat stress (Additional file 5). Among these, *bZIP60* and *bZIP61* were up-regulated 2.92- and 2.91-fold, respectively.

NAC family transcription factors have been implicated in activation of *ERD1* expression [15] and are predominantly induced by abiotic stress in guard cells [16]. Thus, these transcription factors might function in the regulation of photosynthesis under heat stress. Among the 138 NAC family members, 38 were induced under heat stress ranging from 2.06- to 12.23-fold. Expression of *NAC 104* and *NAC 145* was induced 12.15- and 12.23-fold, respectively (Additional file 5).

*MYB* gene family members function in ABA signaling and regulate jasmonic acid-related gene expression, indicating that they affect crosstalk between abiotic and biotic stress responses [17]. In our study, 106 of 178 *MYB* genes were detected as responsive to heat stress. Of these *MYB* genes, 61 were up-regulated, from 2.00- to 39.76-fold. For example, *MYB60*, which promotes stomatal opening, increased by 5.55-fold, but *MYB61*, which promotes stomatal closure, did not increase (Additional file 5).

*CBF/DREB1* activates the expression of genes for osmoprotectants and antioxidants and its expression was quickly and transiently induced by abiotic stress [13]. Of the 60 members of the *DREB* gene family, 42 were differentially expressed in response to high temperature treatment, including 19 up-regulated genes and 23 down-regulated genes. *DREB2*, *DREB7* and *DREB8* were up-regulated 13.9-, 18.26- and 26.42-fold respectively.

*DOF* (DNA binding with One Finger) genes activate expression of photosynthetic genes [36]. In our study, two C2C2-DOF-type TFs (*DOF1* and *DOF-type*) were induced 3.43- and 3.24-fold, respectively.

### Expression of heat-shock transcription factors and heat shock protein genes under heat stress

Heat-shock transcription factors (HSFs) function as key regulators of *APX2* expression in response to oxidative stress caused by excess light [37]. Our microarray data revealed that six *HSF* genes were expressed under high temperature treatment (Additional file 6). Expression of five *HSFs* expression increased, and only *HSFA4C* was repressed under heat stress. *HSFA6B* and *HSF3* showed the highest transcript abundance among the heat-shock transcription factors, and were induced by 135.8-fold and 48.85-fold, respectively, compared with the control group.

Heat shock induces heat shock proteins (HSPs), which play a broad role in many cellular processes, including a generalized function in tolerance to multiple environmental stresses apart from heat stress. In this study, 51 *HSP* genes were differentially expressed in response to heat stress, 44 (86.3%) up-regulated and 7 (13.7%) down-regulated, with expression ratios ranging from 0.029 to 2677 (Figure 8 and Additional file 6). Two *HSP-20 like* genes and one *HSP90* gene were up-regulated more than 1,000-fold under heat stress (Additional file 6). In addition, seven *HSPs* were significantly down-regulated under heat stress. The HSPs can be divided into five classes by molecular weight: small HSPs, (15–30 kDa), HSP40, HSP60, HSP70 and HSP80. The number in each class responding to heat stress was different (Additional file 6). Of the genes responding to heat stress, small heat shock proteins, HSP40 and HSP70 make up the majority, approximately 34.4%, 33% and 15.7% of genes, respectively. Among these genes, *CPN60A* was significantly up-regulated in response to heat stress and the others were significantly down-regulated.

### Verification of microarray data by qRT-PCR

To validate the microarray data, we used qRT-PCR to measure the expression of selected candidate genes representing a variety of functional categories and expression patterns. We focused primarily on transcripts belonging to categories important for photosynthesis related genes. Therefore, we chose 14 genes affecting carbon fixation, electronic transfer and glycollic metabolism and heat responsive transcription factors (Table 3). Comparison of the two methods suggested that real-time PCR revealed the same tendency in changes in expression as the microarray data, despite some differences in expression level. Hence, the results suggest that the microarray data in this study are reliable.

**Figure 9:**
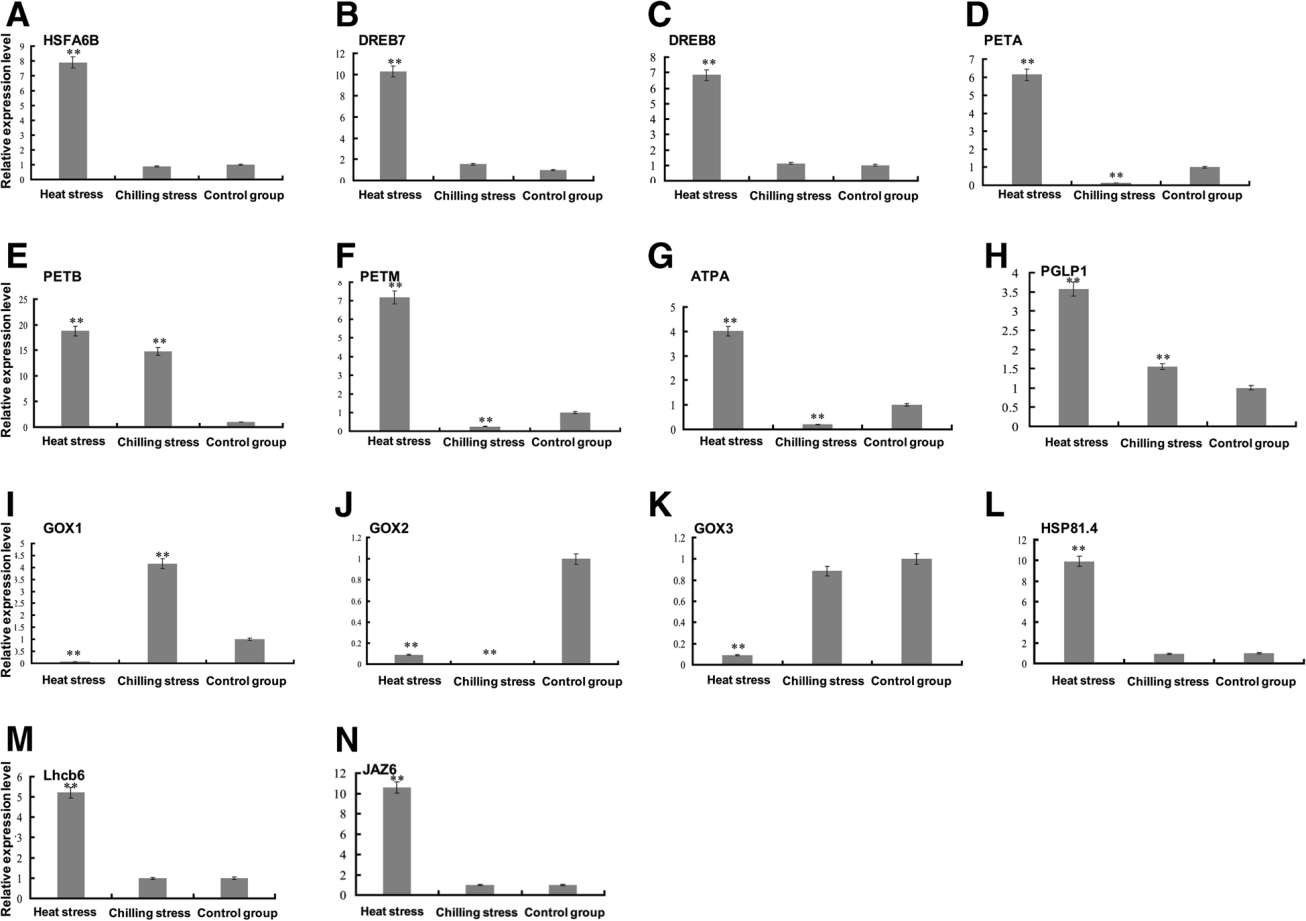
**Quantitative RT-PCR of 14 candidate genes under heat stress, chilling stress, and control conditions. A-N**: represents the expression pattern of *HSFA6B*, *DREB7*, *DREB8*, *PETA*, *PETB*, *PETM*, *ATPA*, *PGLP1*, *GOX1*, *GOX2*, *GOX3*, *HSP81.4*, *Lhcb6* and *JAZ6* genes respectively. Transcript levels are normalized to *PtACTIN* and error bars represent standard error. The control group consisted of three biological samples that were exposure to room temperature under light (25°C, 6 h, 1250 μmolm^-2^s^-1^PPFD). Heat stress indicates samples treated at high temperature under light (42°C, 6 h, 1250 μmolm^-2^s^-1^PPFD). Chilling stress indicates three biological samples that were treated at low temperature under light (4°C, 6 h, 1250 μmolm^-2^s^-1^PPFD) (4°C, 6 h) (‘*’represents *P*-value < 0.05; ‘**’ represents *P*-value < 0.01).

Moreover, we sought to confirm whether these genes were generally temperature-responsive. Therefore, we measured the expression of the 14 candidate genes in response to chilling stress (Figure 9). Nine candidate genes did not respond to chilling stress, including *HSFA6B*, *DREB7*, *DREB8*, *PGLP1*, *GOX2*, *GOX3*, *JAZ*, *HSP81.4*, and *LHCB6*. Only *PETB* showed the same expression tendency under both cold and heat stress. The others showed the opposite tendency of expression under cold and heat stress, including *PETA*, *PETM*, *ATPA* and *GOX1*.

## Discussion

Physiological, biochemical, and transcriptional mechanisms of plants can be affected by high temperature. As the most fundamental physiological process in plants, photosynthesis provides essential energy for plant growth and metabolism [2]. Damage to photosynthesis components may transiently or permanently reduce the overall photosynthetic capacity of a plant [2]. To understand the effects of high temperature on photosynthesis, we measured physiological, biochemical, chlorophyll fluorescence characters and examined changes in the transcriptome in this study.

### Effects of high temperature on photosynthesis

Photosynthesis has been long recognized as sensitive to environment stresses. Pn decreases if environmental stress affects any component of photosynthesis [2]. Our study revealed that photosynthesis significantly decreased after three hours heat stress and subsequently increased at six hours. The main cause of the reduced Pn may be the changes in Gs and Ci [38]. If both Ci and Gs decrease simultaneously, stomatal conductance will mainly limit Pn. By contrast, if Ci increased, but Gs decreased or did not change, the decrease of Pn might be caused by non-stomatal factors. At three hours high temperature treatment, Gs and Ci decreased simultaneously, suggesting that the decreased Pn is mainly caused by stomatal conductance at this time point. Subsequently, a modest increase of Gs and Gi might cause Pn to increase rapidly. After twelve hours of high temperature treatment, Pn, Gs and Gi significantly and simultaneously decreased, suggesting that stomatal conductance again limited Pn in extended heat treatment. Meanwhile, analysis of photosynthesis under heat treatment indicated that photosynthesis completely recovered even six hours treatment. By contrast, after twelve and twenty-four hours of heat treatment, photosynthesis recovered to only 68.8% and 45.2% of control group levels, respectively, implying transient or permanent inhibition. Photosynthesis in plants is composed of interconnected biological processes, including CO_2_ transport and biochemical processes located in the chloroplast thylakoid membranes, stroma, mitochondria and the cytosol of the cell. These biophysical and biochemical processes, and environmental variables determine the net rate of CO_2_ assimilation [24]. Thus, as suggested by Sharkey et al., 2007, presentation of A-Cc curves and a florescence relaxation analysis should be added in future studies to examine how genetics and environment affect photosynthesis.

As a non-intrusive method, chlorophyll fluorescence analysis can detect the effects of environment stress in plants and give insights into the ability of a plant to tolerant environment stresses [39]. Fo is the fluorescence level when all antenna pigment complexes associated with the photosystem are assumed to be open (dark adapted). An increase of Fo represents the extent to which chloroplasts are affected by an environmental stress. Fv/Fm reflects the photosynthetic capability of the entire PSII and the maximum quantum efficiency of open PSII centers [40]. A significant decrease of Fv/Fm suggested an increase in energy dissipation as heat and photoinhibition to the photosynthetic apparatus. *q*P indicated that a percentage of the PSII reaction centers was closed at any time [41]. We found that Fo increased and Fv/Fm, F′v/F′m, *q*P and ETR significantly decreased along with continuous high temperature (42°C), suggesting that the photosystem could be inhibited after 12 h heat stress, together with the limitation of stomatal factors leading Pn, which did not return to normal levels.

### Effects of high temperature on activities of antioxidant enzymes

Temperature stress induced production of reactive oxygen species, which can damage plant cells [42]. To protect the plants from oxidative stress and maintain normal cellular functions, plants have enzymatic scavengers including APX, CAT, POD, SOD, and Glutathione Reductase [43,44]. SOD, as a major scavenger of superoxide anion radicals, provides the first defense mechanism of the antioxidant system [45,46], catalysing the dismutation of O_2_^-^ into H_2_O_2_. Subsequently, CAT, POD and other antioxidant enzymes scavenge H_2_O_2_[47]. Our results indicated that activities of a set of antioxidant enzymes were induced by high temperature stress at three and six hours, implying that the combined action of SOD, CAT, POD and APX converts the toxic O_2_^-^ and H_2_O_2_ to water and molecular oxygen (O_2_), thereby protecting the cell from oxidative stress. At twelve and twenty-four hours, all of the activities of antioxidant enzymes were repressed, suggesting that the efficiency of scavenging O_2_^-^ and H_2_O_2_ might be decreased, thus damaging cellular membranes.

MDA concentrations indicate the extent of lipid peroxidation caused by oxidative stress [48]. In this study, the progressive high temperature stress resulted in MDA concentrations that sharply increased after twelve hours of heat stress, indicating that membrane damage had occurred. Also, H_2_O_2_ increased significantly. Combined with activities of antioxidants enzymes suggesting that efficiency of scavenging O_2_^-^ and H_2_O_2_ decreased along with decreases in activities of antioxidants enzymes, leading to damage in cellular membrane after twelve hours of heat treatment.

### Heat-responsive genes involved in photosynthesis

Efficient photosynthesis involves photosynthetic pigments and photosystems, the electron transport system, CO_2_ fixation pathways, and glycollic metabolism. Damage to any of these components may reduce photosynthetic capacity [2]. It has been long believed that the major heat-sensitive component is the PSII center [49]. Consistent with this conclusion, our results revealed that 20 differentially expressed genes were detected for PSII, with only four genes increased and 16 genes decreased. More genes were down-regulated than up-regulated, suggesting that PSII might be suffered more negative effects from heat stress than PSI.

The majority of photosynthetic energy is harnessed via linear electron flow involving light-stimulated electron transfer between two reaction centers, PSI and PSII [50]. Interestingly, in the light reaction, all four genes (*PETA*, *PETB*, *PETM* and *ATPA*) for the redox chain were up-regulated at six hours, implying that electron transport might be induced by high temperature at this timepoint. Sharkey et al. (2005) reported that the cyclic transport of electrons can be induced by moderately high temperature (35°C - 45°C) and thylakoid membranes become leaky at the same time [5]. Tozzi et al. (2013) suggested that significant increases in the rate of cyclic electron transport at high temperatures may counteract thylakoid membrane leakiness and provide protection against irreversible damage. In contrary, Ferreira et al. (2006) indicated that photosynthetic linear electron flow was induced along with a decrease of PSII abundance and an increase of PSI in *P. euphratica*[14]. These suggest that electron transport (cyclic or linear) induced by heat stress in *P. simonii* needs further study.

In photosynthesis, *PSBD* encodes PSII D2, which produces non-radiative energy dissipation, a highly effective protective mechanism against photodamage. Sane et al. (2002) indicated that the accumulation of PSII D2 protein may promote resistance to high excitation pressure induced by exposure to either low temperature or high light [51]. Our data showed that *PSBD* was significantly up-regulated at six hours of heat treatment, suggesting that *PSBD* might be involved in protective mechanisms against photodamage at this time point. Cytochrome b6f mediates the transfer of electrons between the two photosynthetic reaction centers, while protons are transferred from the chloroplast stroma across the thylakoid membrane into the lumen [52]. Electron transport via cytochrome b6f creates the proton gradient that drives the synthesis of ATP in chloroplasts, which is essential for repair of PSII [53]. *PETA, PETB*, and *PETM*, encoding cytochrome a, b and m(6) subunits of the cytochrome b6f complex respectively, were up-regulated significantly under heat stress compared with controls, suggesting that *PETB* in *P. simonii* may play an important role in adenosine triphosphate (ATP) production and repair of PSII under heat stress. Meanwhile, *ATPA*, encoding the ATPase alpha subunit, which catalyzes the conversion of ADP to ATP using the proton motive force, was up-regulated 5.01-fold under heat stress. Gene expression results revealed that the combined action of these four genes promotes the synthesis of ATP under high temperature.

In the Calvin cycle, seven genes involved in carboxylation, reduction and regeneration were significantly repressed, suggesting that these processes were negatively regulated by heat stress (Table 2 and Figure 6). It is fairly well known Rubisco activase enzyme catalyzes the carboxylation of ribulose-1, 5-bisphosphate for fixation of CO_2_ in photosynthesis and its denaturing/disruption occurs at roughly 35 - 38°C [54]. It is suggesting that the represses of carboxylation processes is likely a cause for the Rubisco decline that causes photosynthesis to plummet at higher temperatures. As suggested by Sharkey and Zhang (2010), Rubisco deactivation may be a protective acclimation strategy for heat tolerance [55]. By contrast, *CPN60A*, which is involved in carboxylation, was up-regulated under heat stress. CPN60 plays an important role in protecting plant photosynthesis against heat stress and also affects the recovery of photosynthesis [56]. *CPN60A* was significantly up-regulated under heat stress, indicating that the mechanisms for protection of photosynthesis were activated in *P. simonii* under heat stress.

As a key role in CO_2_ fixation, Rubisco is not completely capable of discriminating its substrate CO_2_ and O_2_ during oxygenic photosynthesis. Thus, 2-phosphoglycolate (2-PG) is produced by oxygenation of RuBP, a strong inhibitor of enzymes in photosynthetic carbon metabolism [57,58]. 2-PG can be scavenged by photorespiration and converted to 3-phosphoglycerate, which can re-enter the Calvin cycle [59,60]. The chloroplast enzyme PGLP1 catalyzes the first reaction of the photorespiratory C2 cycle that converts 2-phosphoglycolate to glycolate [61]. In our study, *PGLP1*, encoding 2-phosphoglycolate phosphatase, was persistently up-regulated with heat stress suggesting that the inhibition of photosynthesis was released due to scavenging of 2-PG. This might lead to increases in glycolate concentrations, along with 2-phosphoglycolate metabolism. The photorespiratory enzyme GOX plays an important role in converting glycolate to glyoxylate and in H_2_O_2_ production [62]. Three members of the *GOX* gene family were differentially expressed under heat stress. At six hours, all three *GOX* genes were down-regulated, suggesting that the conversion of glycolate to glyoxylate was inhibited and H_2_O_2_ was not produced. After twelve hours of high temperature treatment, the abundance of *PGLP1* and *GOX1-3* transcripts were significantly and consistently increased, suggesting that regulation of these genes, combined with decreases in activities of antioxidant enzymes, might be the main reason for massive production of H_2_O_2_.

After three hours of heat treatment and recovery at room temperature, the expression of all eight candidate genes recovered to control levels, suggesting that plant photosynthesis was not damaged by three hours of heat treatment. Among these genes, *PETM* and *PETB* gene expression were higher than controls after recovery from 6h heat treatment, implying that moderately high temperature might help ETR, consistent with Sharkey et al. (2005) [5]. After recovery from twelve hours of heat treatment, expression of all ETR-related genes did not recover completely, suggesting that irreversible damage was caused by prolonged heat stress. Differently, expression of *PGLP1* and *GOX1-3* was higher than the control group under heat stress of more than six hours, implying that the production of H_2_O_2_ might be maintained for a long time. This is consistent with the H_2_O_2_ measurements after recovery from heat stress. H_2_O_2_ is often seen as part of the plant signaling cascade leading to protection from abiotic stresses; therefore *PGLP1* and *GOX1-3* expression might improve poplar stress adaptation [63].

### Heat-responsive transcription factors

The DREB family is important in regulating plant responses to abiotic stress. DREBs belong to the AP2/ERF family of transcription factors (Yamaguchi-Shinozaki and Shinozaki 1994), which confer stress tolerance in plants and are one of the largest and most diverse families of proteins involved in the regulation of plant responses [64]. In *Arabidopsis*, there are two classes of *DREB* genes, *DREB1* and *DREB2*[65]. *DREB2* genes were initially identified as drought and high-salinity response genes [66], and were later shown to be induced in response to heat stress [67,68]. However, in this study, only three *DREB2* genes were detected and found to be significantly up-regulated under heat stress, suggesting that, in contrast to *DREB2*, in poplar, *DREB1* might not respond to heat stress.

The result that DREBs distinguish cold and dehydration signal transduction pathways was consistent with previous studies. For example, Chen et al. (2010) identified heat shock transcription factor A3 (*HsfA3*) as a highly up-regulated heat-inducible gene in transgenic plants over-expressing *DREB2C*[69]. Moreover, *HsfA3* expression is directly regulated by *DREB2s* under heat stress [69,70] and *DREB2*-overexpressing transgenic plants have increased tolerance to heat stress [71,72]. DREB2C interacts with ABF2, a bZIP protein regulating ABA-responsive gene expression, and its overexpression is affected ABA sensitivity [72]. Hwang et al. (2012) found that *DREB2C* plays an important role in promoting oxidative stress tolerance in *Arabidopsis*, suggesting that DREB2Cs may function as multi stimuli-response factors that interact with genes and/or proteins during different stress conditions [73,74]. In our study, we found that *HSFA3* and *DREB2C* were significantly up-regulated under heat stress, providing indirect evidence for interaction of *HSFA3* and *DREB2C.*

The *STRESS-RESPONSIVE NAC1* (*SNAC1*) gene is predominantly induced by abiotic stress in guard cells [16]. Over-expression of *SNAC1* in plants did not produce a negative phenotype, unlike overexpression of *CBF/DREB1*[75]. Overexpression of *SNAC1* increased abiotic stress tolerance, reduced transpiration rate and increased ABA sensitivity. Our results indicated that *NAC001* was significantly up-regulated after six hours of heat stress with notably increased stomatal closure, suggesting that it plays a key role in regulating stomatal dynamics in poplar under heat stress.

*MYB60* and *MYB61* are directly involved in stomatal dynamics in *Arabidopsis* and play opposite roles in stomatal closure: *MYB60* promotes stomatal opening, and *MYB 61* promotes stomatal closure [76,77]. In rice, the over-expression of *SNAC1* up-regulates *MYB61*[16]. In this study, *MYB61* was not expressed, suggesting that the regulatory relationship of *NAC1* and *MYB61* is still unclear. *MYB60* expression was significantly up-regulated, suggesting that it might play a key role in stomatal conductance from three to six hours of heat stress.

*DOF1*, as an activator of transcription, can regulate photosynthesis-related genes in accumulation of grain proteins and affect yield through regulation of nitrogen metabolism [78,79]). Over-expression of *DOF1* enhances expression of genes associated with carbon skeleton production [80]. In our study, *DOF1* was significantly up-regulated under heat stress, suggesting carbon fixation might not be repressed under six hours of heat stress.

## Conclusion

This study provides a systematic physiological and global expression profile of the response of poplar photosynthetic to heat stress. Analysis of photosynthesis under heat treatment indicated that stomatal conductance is the main cause for the decrease in Gs and Ci at three hours, and continuing after twelve hours, of high temperature treatment. Over twelve hours high temperature treatment might cause permanent inhibition of photosynthesis. Chlorophyll fluorescence analysis showed that photosystems could be inhibited after twelve hours heat stress, together with the limitation of stomatal factors altering Pn, which did not completely return to normal levels. Combined photosynthetic physiology and gene expression analyses indicated that ETR, in the light reaction, was not significantly changed, but expression of four genes (*PETA*, *PETB*, *PETM* and *ATPA*) for the redox chain was up-regulated at six hours heat stress, implying that cyclic electron transport might be induced by high temperature at this time point. In the Calvin cycle, three genes involved in carboxylation were significantly repressed, suggesting that repression of carboxylation processes is the likely cause for the decline in Rubisco activity, which causes photosynthesis to plummet at higher temperatures.

Our physiological results showed that twelve hours heat stress is a threshold for the combined action of antioxidant enzymes that convert the toxic O_2_^-^ and H_2_O_2_ to water and O_2_, thereby protecting the cell from oxidative stress. Meanwhile, the abundance of *PGLP1* and *GOX1-3* transcripts significantly and consistently increased, along with decreases in activities of antioxidant enzymes, which might be the main reason for massive production of H_2_O_2_. Also, expression of *PGLP1* and *GOX1-3* was higher than the control group under heat stress of more than six hours, implying that the production of H_2_O_2_ might be maintained for a long time. H_2_O_2_ has important functions in plant signaling cascades leading to protection from abiotic stresses; therefore *PGLP1* and *GOX1-3* might be important candidate genes for improving poplar stress adaptation in future research.

The genome-wide gene expression analysis conducted in this study detected a set of heat-responsive transcription factors, which included *HSFA3*, *DREB2C, NAC1, MYB 60* and *DOF1*. These genes play important roles in regulating stomatal dynamics and heat stress responses in poplar under high temperature treatment. Thus, the photosynthetic physiology and gene expression analyses of this study have expanded our understanding of plant thermostability and will help identify candidate genes that regulate the heat stress response of poplar.

## Abbreviations

ATP: Adenosine triphosphate; ATPA: A-subunit of the coupling-factor-1 (CF1) ATP synthase; AOAT2: Alanine-2-oxoglutarate aminotransferase 2; bZIP: Basic leucine zipper; CAT: Catalase; CBF/DREB1: C repeat binding factor/drought response element binding 1; Ci: Intercellular CO_2_ concentration; CPN60A: Chaperonin-60ALPHA; DOF: DNA binding with one finger; ETR: Electron transport rate; ERD1: Early responsive to dehydration stress 1; Fo: minimum fluorescence; Fm: maximum fluorescence; Fv: Variable fluorescence; F'o: The minimum fluorescence; F'v: Variable fluorescence; F'm: Maximum fluorescence; Fs: Steady state parameter; GDCST: T-Protein of the glycine; GOX1: Glycolate oxidase 1; GOX2: Glycolate oxidase 2; GOX3: Glycolate oxidase 3; Gs: Stomatal conductance; H2DCF-DA: H_2_O_2_-specific fluorescent probe 2',7'-Dichlorodihydrofluorescein diacetate; HSFS: Heat-shock transcription factors; HSFA3: Heat shock transcription factor A3; iWUE: Intrinsic water use efficiency; MDA: Malondialdehyde; NAC: NAM, ATAF1/2, CUC2; PETA: Photosynthetic electron transport A; PETM: Photosynthetic electron transport M; PETB: Photosynthetic electron transport B; PGLP1: Phosphoglycolate phosphatase 1; Pn: net photosynthetic rate; POD: peroxidase; qP: Photochemical quenching; qPCR: Quantitative real-time polymerase chain reaction; RCA: Ribulose bisphosphate carboxylase/oxygenase activase; SBP: Squamosa promoter binding proteins; SNAC1: Stress-responsive NAC1; SOD: Superoxide dismutase; Tr: transpiration rate; TFs: Transcription factors.

## Competing interests

The authors declared that they have no competing interests.

## Authors’ contributions

Conceived and designed the experiments: DZ. Performed the experiments: YS, QC, QC, XS, and DZ. Analyzed the data: YS, QC, XS, DC, and DZ. Contributed reagents/materials/analysis tools: DZ. Wrote the paper: YS, QC, and, DZ. All authors read and approved the final manuscript.

## Supplementary Material

Additional file 1 Real-time PCR primer sequences. Click here for file

Additional file 2 **Poplar *ACTINII-like*** **gene (Accession number: EF145577) has stable expression under high temperature treatment and was used as the internal control.** Click here for file

Additional file 3 GO terms of genes up-regulated under heat stress. Click here for file

Additional file 4 GO terms of genes down-regulated under heat stress. Click here for file

Additional file 5 Candidate transcription factors. Click here for file

Additional file 6 Differentially expressed HSF and HSP genes. Click here for file

## Accession numbers

The gene expression data reported here are available from NCBI with the GEO accession number GSE41557.
