# Supplementary material for: Effects of high temperature on photosynthesis and related gene expression in poplar
Source: BMC Plant Biol. 2014 Apr 28;14:111. doi: 10.1186/1471-2229-14-111 (PMC4036403; doi:10.1186/1471-2229-14-111)
Supplement: Additional file 1 — Real-time PCR primer sequences. [file 1471-2229-14-111-S1.doc]

**Additional 1 Real-time PCR primer sequences**

| Primer name | Primer sequences(5′-3′) | Size (bp) | *Tm* (°C) | Efficiency (%) |
| --- | --- | --- | --- | --- |
| Potri.005G214800 | F:ATTGGGTAGCGAAGGTGAAGAC  R:ACCCAAGTAACCAAGCTGTTCAA | 67 | 58.0 | 98.9~105.4 |
| Potri.010G183700 | F:GGACCAATGACTGGCACAACT  R:CCCCTAGCAACTCATCAAAGCT | 63 | 58.0 | 97.2~103.1 |
| Potri.008G073600 | F:AAGGTAAAGGAGGGCCAGAGAA  R:CCCCATGTCCTCTGTCTCACA | 64 | 58.0 | 95.9~104.9 |
| Potri.T058600 | F:CCTCCCAGGTTGCTCATCA  R:ACAGTAGGGAACGGAGCTGAAG | 60 | 58.0 | 98.8~105.6 |
| Potri.004G003000 | F:CCGTTATCTCCACATCCTTCATT  R:TGTCGACCCTCTCTCCTTCAA | 68 | 58.0 | 97.5~100.7 |
| Potri.013G137300 | F:GTGCTGCAATTGCTGCCTTAT  R:GGAAGGATCCAAGGGATTTAACA | 72 | 58.0 | 98.6~101.2 |
| Potri.013G138000 | F:TGAGCGTGAAGGGTTCAAAGA  R:CAGTTTTGGCCCCAGTTACG | 61 | 58.0 | 97.8~104.3 |
| Potri.008G077400 | F:TCACCTGGTTCGCTCAAAAAT  R:GGTAGAGGTGCGCCATCGT | 64 | 58.0 | 99.7~108.1 |
| Potri.001G394400 | F:TCGTGTGTCACATTTGGCAGTA  R:GCCTGGTAACGATCCTCTTGTT | 78 | 58.0 | 99.1~101.9 |
| Potri.002G027000 | F:ACCGGAACCGGAAGTGACT  R:TTGCCAATATCAAACACTCATCCT | 62 | 58.0 | 97.9~102.1 |
| Potri.011G112700 | F:CGGTGGGTGGATTTCTTCAA  R:CATGGTGTCGCCCATTCC | 56 | 58.0 | 98.2~105.2 |
| Potri.003G020400 | F:CGCGGCTGGAGAAGAAGAT  R:TTTGGCTGCTCATTAATCAGCTT | 61 | 58.0 | 99.3~100.9 |
| Potri.003G068900 | F:CCTAGGCCTCCCAGATCTGAA  R:TCTGATGATTGGCCTTCGTCTA | 59 | 58.0 | 99.9~106.8 |
| Potri.016G003400 | F:TTGCTTTTCGAGACAGCTATGC  R:CAAAGGTGTTGGGATCATCCA | 61 | 58.0 | 98.8~103.2 |
