# Supplementary material for: Effects of high temperature on photosynthesis and related gene expression in poplar
Source: BMC Plant Biol. 2014 Apr 28;14:111. doi: 10.1186/1471-2229-14-111 (PMC4036403; doi:10.1186/1471-2229-14-111)
Supplement: Additional file 2 — Poplar ACTINII-like gene (Accession number: EF145577) has stable expression under high temperature treatment and was used as the internal control. [file 1471-2229-14-111-S2.doc]

0h

3h

6h

12h

24h

**
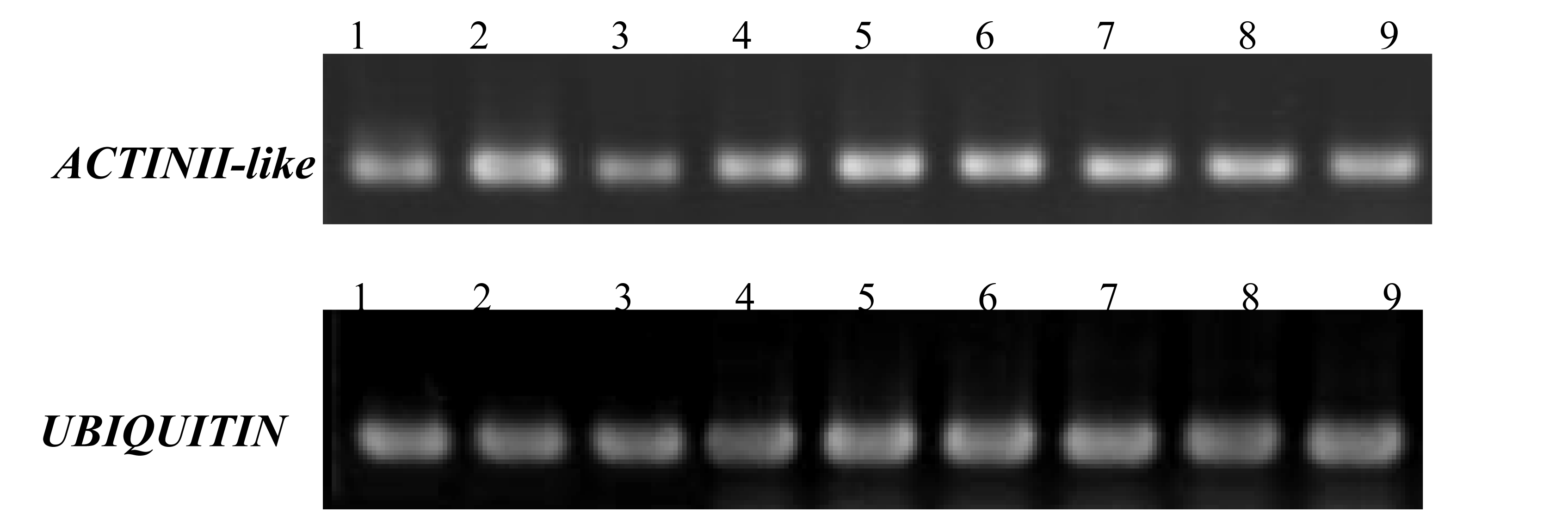
**

**Additional file 2** Poplar *ACTINII-like* gene (Accession number: EF145577) has stable expression under high temperature treatment and was used as the internal control. 0h indicates control group without high temperature treatment. 3-24h indicates different times of exposure to heat stress.
