# Supplementary material for: Effects of high temperature on photosynthesis and related gene expression in poplar
Source: BMC Plant Biol. 2014 Apr 28;14:111. doi: 10.1186/1471-2229-14-111 (PMC4036403; doi:10.1186/1471-2229-14-111)
Supplement: Additional file 3 — GO terms of genes up-regulated under heat stress. [file 1471-2229-14-111-S3.doc]

**Additional file 3 GO terms of genes up-regulated under heat stress**

| GO term Ontology | Ontology | Description | Number in input list | Number in BG/Ref | p-value | FDR |
| --- | --- | --- | --- | --- | --- | --- |
| GO:0060590 | F | ATPase regulator activity | 6 | 12 | 1.7e-08 | 3.3e-06 |
| GO:0051087 | F | chaperone binding | 6 | 12 | 1.7e-08 | 3.3e-06 |
| GO:0031072 | F | heat shock protein binding | 14 | 162 | 6.5e-07 | 8.3e-05 |
| GO:0003735 | F | structural constituent of ribosome | 23 | 434 | 1.7e-06 | 0.00016 |
| GO:0005198 | F | structural molecule activity | 24 | 507 | 6.8e-06 | 0.00052 |
| GO:0051082 | F | unfolded protein binding | 6 | 59 | 0.00043 | 0.027 |
| GO:0005737 | C | cytoplasm | 59 | 1165 | 3.3e-13 | 4e-11 |
| GO:0044444 | C | cytoplasmic part | 46 | 938 | 2.8e-10 | 1.7e-08 |
| GO:0005739 | C | mitochondrion | 15 | 124 | 2.9e-09 | 1.1e-07 |
| GO:0030529 | C | ribonucleoprotein complex | 26 | 476 | 2.1e-07 | 6.3e-06 |
| GO:0044424 | C | intracellular part | 77 | 2492 | 6e-07 | 1.4e-05 |
| GO:0044429 | C | mitochondrial part | 11 | 100 | 9.3e-07 | 1.9e-05 |
| GO:0005840 | C | ribosome | 23 | 434 | 1.7e-06 | 2.8e-05 |
| GO:0005622 | C | intracellular | 92 | 3314 | 4.6e-06 | 6.9e-05 |
| GO:0043232 | C | intracellular non-membrane-bounded organelle | 25 | 600 | 3.7e-05 | 0.00044 |
| GO:0043228 | C | non-membrane-bounded organelle | 25 | 600 | 3.7e-05 | 0.00044 |
| GO:0031974 | C | membrane-enclosed lumen | 7 | 60 | 6e-05 | 0.00057 |
| GO:0043229 | C | intracellular organelle | 56 | 1910 | 6.1e-05 | 0.00057 |
| GO:0043226 | C | organelle | 56 | 1910 | 6.1e-05 | 0.00057 |
| GO:0005740 | C | mitochondrial envelope | 8 | 89 | 0.00012 | 0.001 |
| GO:0032991 | C | macromolecular complex | 36 | 1145 | 0.0003 | 0.0022 |
| GO:0044464 | C | cell part | 131 | 5684 | 0.00031 | 0.0022 |
| GO:0005623 | C | cell | 131 | 5684 | 0.00031 | 0.0022 |
| GO:0031967 | C | organelle envelope | 8 | 112 | 0.00057 | 0.0038 |
| GO:0031975 | C | envelope | 8 | 142 | 0.0026 | 0.017 |
| GO:0044422 | C | organelle part | 15 | 446 | 0.0083 | 0.048 |
| GO:0044446 | C | intracellular organelle part | 15 | 446 | 0.0083 | 0.048 |

F represents molecular function; C represents cellular component
