# Supplementary material for: Effects of high temperature on photosynthesis and related gene expression in poplar
Source: BMC Plant Biol. 2014 Apr 28;14:111. doi: 10.1186/1471-2229-14-111 (PMC4036403; doi:10.1186/1471-2229-14-111)
Supplement: Additional file 4 — GO terms of genes down-regulated under heat stress. [file 1471-2229-14-111-S4.doc]

**Additional file 4 GO terms of genes down-regulated under heat stress**

| GO term Ontology | Ontology | Description | Number in input list | Number in BG/Ref | p-value | FDR |
| --- | --- | --- | --- | --- | --- | --- |
| GO:0003824 | F | catalytic activity | 313 | 9307 | 2.2e-09 | 1.1e-06 |
| GO:0016787 | F | hydrolase activity | 110 | 2834 | 3.2e-07 | 8.1e-05 |
| GO:0043169 | F | cation binding | 92 | 2449 | 8.2e-06 | 0.00083 |
| GO:0046872 | F | metal ion binding | 92 | 2445 | 7.7e-06 | 0.00083 |
| GO:0043167 | F | ion binding | 92 | 2449 | 8.2e-06 | 0.00083 |
| GO:0008270 | F | zinc ion binding | 54 | 1259 | 1.7e-05 | 0.0014 |
| GO:0003700 | F | transcription factor activity | 38 | 805 | 3.8e-05 | 0.0027 |
| GO:0003677 | F | DNA binding | 73 | 1929 | 4.7e-05 | 0.0029 |
| GO:0046914 | F | transition metal ion binding | 76 | 2041 | 5.6e-05 | 0.0031 |
| GO:0030528 | F | transcription regulator activity | 51 | 1244 | 8.5e-05 | 0.0043 |
| GO:0016798 | F | hydrolase activity, acting on glycosyl bonds | 26 | 505 | 0.00015 | 0.0063 |
| GO:0004553 | F | hydrolase activity, hydrolyzing O-glycosyl compounds | 25 | 477 | 0.00015 | 0.0063 |
| GO:0016491 | F | oxidoreductase activity | 68 | 1867 | 0.00023 | 0.0089 |
| GO:0005488 | F | binding | 309 | 10853 | 0.00036 | 0.012 |
| GO:0016762 | F | xyloglucan:xyloglucosyl transferase activity | 6 | 42 | 0.00037 | 0.012 |
| GO:0043565 | F | sequence-specific DNA binding | 27 | 578 | 0.00051 | 0.016 |
| GO:0005634 | C | nucleus | 43 | 975 | 5.9e-05 | 0.0065 |
| GO:0030312 | C | external encapsulating structure | 12 | 166 | 0.00049 | 0.013 |
| GO:0048046 | C | apoplast | 6 | 42 | 0.00037 | 0.013 |
| GO:0005618 | C | cell wall | 11 | 136 | 0.00032 | 0.013 |
| GO:0005576 | C | extracellular region | 7 | 77 | 0.0019 | 0.031 |
| GO:0044464 | C | cell part | 166 | 5684 | 0.002 | 0.031 |
| GO:0005623 | C | cell | 166 | 5684 | 0.002 | 0.031 |
| GO:0043231 | C | intracellular membrane-bounded organelle | 48 | 1342 | 0.0022 | 0.031 |
| GO:0043227 | C | membrane-bounded organelle | 48 | 1354 | 0.0027 | 0.032 |

F represents molecular function; C represents cellular component
