# Supplementary material for: Effects of high temperature on photosynthesis and related gene expression in poplar
Source: BMC Plant Biol. 2014 Apr 28;14:111. doi: 10.1186/1471-2229-14-111 (PMC4036403; doi:10.1186/1471-2229-14-111)
Supplement: Additional file 5 — Candidate transcription factors. [file 1471-2229-14-111-S5.pdf]

Additional file 5 Candidate transcription factors

Probe	Foldchange	Poplar Gene model	TAIR gene model	Annotation
PtpAffx.26464.1.A1_at	135.7996034	POPTR_0005s23640	AT3G22830	member of Heat Stress Transcription Factor (Hsf) family
PtpAffx.118187.1.A1_s	48.85299699	POPTR_0006s11680	AT5G03720	Member of Heat Stress Transcription Factor (Hsf) family. Expression is regulated by DREB2A and in turn HSFA3 regulates the expression of hsps Hsp18.1-CI and Hsp26.5-MII35S. Involved in establishing thermotolerence.
PtpAffx.37260.1.S1_at	27.57623052	POPTR_0018s06080	AT3G24500	One of three genes in A. thaliana encoding multiprotein bridging factor 1, a highly conserved transcriptional coactivator. May serve as a bridging factor between a bZIP factor and TBP. Its expression is specifically elevated in response
PtpAffx.92099.1.A1_s_	26.42320012	POPTR_0008s07360	AT2G40340	Encodes a member of the DREB subfamily A-2 of ERF/AP2 transcription factor family. The protein contains one AP2 domain. There are eight members in this subfamily including DREB2A AND DREB2B that are involved in response
PtpAffx.47307.1.A1_at	21.267537	POPTR_0007s07830	AT1G56170	Encodes a protein with similarity to a subunit of the CCAAT promoter motif binding complex of yeast.One of two members of this class (HAP5B) and expressed in vegetative and reproductive tissues
PtpAffx.209249.1.S1_a	18.26485899	POPTR_0010s19100	AT2G40340	Encodes a member of the DREB subfamily A-2 of ERF/AP2 transcription factor family. The protein contains one AP2 domain. There are eight members in this subfamily including DREB2A AND DREB2B that are involved in response
PtpAffx.59348.1.S1_at	14.15634975	POPTR_0003s06670	AT1G72450	JAZ6 transcript levels rise in response to a jasmonate stimulus and a GFP:JAZ6 fusion protein localizes to the nucleus. Application of jasmonate methyl ester to Arabidopsis roots reduces the levels of a JAZ6:GUS fusion protein, presun
PtpAffx.69785.2.A1_a_	11.76774384	POPTR_0016s08600	AT2G37060	nuclear factor Y, subunit B8 (NF-YB8); FUNCTIONS IN: sequence-specific DNA binding transcription factor activity; INVOLVED IN: regulation of transcription, DNA-dependent; LOCATED IN: nucleus, intracellular; EXPRESSED
PtpAffx.205263.1.S1_x	11.07717828	POPTR_0005s09770	AT1G56170	Encodes a protein with similarity to a subunit of the CCAAT promoter motif binding complex of yeast.One of two members of this class (HAP5B) and expressed in vegetative and reproductive tissues
PtpAffx.121305.1.A1_a	11.03716141	POPTR_0007s15240	AT1G78930	Mitochondrial transcription termination factor family protein; FUNCTIONS IN: molecular_function unknown; INVOLVED IN: biological_process unknown; LOCATED IN: chloroplast; EXPRESSED IN: 22 plant structures; EXPRESS
PtpAffx.89026.1.A1_at	10.84851691	POPTR_0006s26500	AT5G11340	Acyl-CoA N-acyltransferases (NAT) superfamily protein; FUNCTIONS IN: N-acetyltransferase activity; INVOLVED IN: metabolic process; LOCATED IN: cellular_component unknown; EXPRESSED IN: 23 plant structures; EXPRE
PtpAffx.23267.1.A1_at	10.6528486	POPTR_0019s12530	AT1G05710	basic helix-loop-helix (bHLH) DNA-binding superfamily protein; FUNCTIONS IN: sequence-specific DNA binding transcription factor activity; INVOLVED IN: response to ethylene stimulus, regulation of transcription; LOCATED IN
Ptp.1156.1.S1_at	10.57315499	POPTR_0002s16460	AT5G52660	Homeodomain-like superfamily protein; CONTAINS InterPro DOMAIN/s: SANT, DNA-binding (InterPro:IPR001005), Homeodomain-like (InterPro:IPR009057), Myb, DNA-binding (InterPro:IPR014778), HTH transcriptional regulat
PtpAffx.210750.1.S1_a	9.742548375	POPTR_0012s13430	AT5G62020	member of Heat Stress Transcription Factor (Hsf) family
PtpAffx.11878.2.S1_at	8.056064235	POPTR_0005s18430	AT5G67190	encodes a member of the DREB subfamily A-5 of ERF/AP2 transcription factor family. The protein contains one AP2 domain. There are 16 members in this subfamily including RAP2.1, RAP2.9 and RAP2.10.
Ptp.6756.1.S1_at	7.455497019	POPTR_0007s02510	AT2G46410	Nuclear-localized R3-type MYB transcription factor. Positive regulator of hair-cell differentiation. Preferentially transcribed in hairless cells. Moves from atrichoblasts into trichoblast via plasmodesmata in a tissue-specific mode. N-term
PtpAffx.145393.1.A1_a	7.242967425	POPTR_0010s16130	AT1G48040	Protein phosphatase 2C family protein; FUNCTIONS IN: protein serine/threonine phosphatase activity, catalytic activity; INVOLVED IN: protein amino acid dephosphorylation; LOCATED IN: protein serine/threonine phosphatase com
PtpAffx.95473.1.A1_at	6.762513169	POPTR_0002s08150	AT1G01720	Belongs to a large family of putative transcriptional activators with NAC domain. Transcript level increases in response to wounding and abscisic acid. ATAF1 attenuates ABA signaling and sythesis. Mutants are hyposensitive to ABA.
PtpAffx.202952.1.S1_a	6.724796163	POPTR_0003s05100	AT1G79840	Glabra 2, a homeodomain protein affects epidermal cell identity including trichomes, root hairs, and seed coat. It also down-regulates seed oil content. Expressed in atrichoblasts and required to suppress root hair development. Also expre
PtpAffx.13229.3.A1_at	6.712201179	POPTR_0006s23390	AT5G20900	jasmonate-zim-domain protein 12 (JAZ12); FUNCTIONS IN: protein binding; INVOLVED IN: biological_process unknown; LOCATED IN: cellular_component unknown; EXPRESSED IN: 25 plant structures; EXPRESSED DURINC
PtpAffx.5686.2.S1_at	6.674484924	POPTR_0011s10670	AT1G78600	light-regulated zinc finger protein 1 (LZF1); CONTAINS InterPro DOMAIN/s: Zinc finger, B-box (InterPro:IPR000315); BEST Arabidopsis thaliana protein match is: B-box zinc finger family protein (TAIR:AT1G06040.1); Has 1826 E
PtpAffx.203182.1.S1_a	6.640832573	POPTR_0003s11420	AT4G12240	zinc finger (C2H2 type) family protein; CONTAINS InterPro DOMAIN/s: Zinc finger, C2H2-like (InterPro:IPR015880), Zinc finger, C2H2-type (InterPro:IPR007087); BEST Arabidopsis thaliana protein match is: C2H2-like zinc finge
PtpAffx.212769.1.S1_a	6.429052047	POPTR_0015s08580	AT4G20970	basic helix-loop-helix (bHLH) DNA-binding superfamily protein; FUNCTIONS IN: DNA binding, sequence-specific DNA binding transcription factor activity; INVOLVED IN: defense response to fungus, regulation of transcription; L
PtpAffx.203919.1.S1_a	6.254643359	POPTR_0004s09920	AT1G67100	LOB domain-containing protein 40 (LBD40); CONTAINS InterPro DOMAIN/s: Lateral organ boundaries, LOB (InterPro:IPR004883); BEST Arabidopsis thaliana protein match is: LOB domain-containing protein 41 (TAIR:AT3G025
PtpAffx.159340.1.A1_a	6.209047248	POPTR_0001s19070	AT3G16350	Homeodomain-like superfamily protein; CONTAINS InterPro DOMAIN/s: SANT, DNA-binding (InterPro:IPR001005), Homeodomain-like (InterPro:IPR009057), Myb, DNA-binding (InterPro:IPR014778), Zinc finger, CCHC-type (In
PtpAffx.30938.1.A1_s_	6.160836147	POPTR_0007s11030	AT4G36990	encodes a protein whose sequence is similar to heat shock factors that regulate the expression of heat shock proteins. Transcript level is increased in response to heat shock. However, overexpression of this gene did not result in the increa
Ptp.7035.1.S1_at	5.715918765	POPTR_0018s01280	AT5G11340	Acyl-CoA N-acyltransferases (NAT) superfamily protein; FUNCTIONS IN: N-acetyltransferase activity; INVOLVED IN: metabolic process; LOCATED IN: cellular_component unknown; EXPRESSED IN: 23 plant structures; EXPRE
PtpAffx.123170.1.S1_a	5.65332002	POPTR_0015s10940	AT5G63470	nuclear factor Y, subunit C4 (NF-YC4); CONTAINS InterPro DOMAIN/s: Transcription factor CBF/NF-Y/archaeal histone (InterPro:IPR003958), Histone-fold (InterPro:IPR009072); BEST Arabidopsis thaliana protein match is: nucle
PtpAffx.16157.1.S1_s_	5.59382511	POPTR_0006s10240	AT5G01380	Homeodomain-like superfamily protein; CONTAINS InterPro DOMAIN/s: SANT, DNA-binding (InterPro:IPR001005), MYB-like (InterPro:IPR017877); BEST Arabidopsis thaliana protein match is: Homeodomain-like superfamily pr
PtpAffx.224943.1.S1_s	5.551326429	POPTR_0019s05190	AT3G13540	Encodes a member of the MYB family of transcriptional regulators. MYB5 act as a negative regulator of trichome branching and play a role in the correct formation of the seed coat and possibly the formation the underlying endosperm l
PtpAffx.18907.1.A1_s_	5.39065567	POPTR_0007s11470	AT4G37180	myb family transcription factor, contains Pfam domain, PF00249: Myb-like DNA-binding domain l; also isolated as a putative cytoskeletal protein in a yeast screen
PtpAffx.215822.1.S1_s	5.181187117	POPTR_0004s14790	AT3G19290	bZIP transcription factor with specificity for abscisic acid-responsive elements (ABRE). Mediate ABA-dependent stress responses.
PtpAffx.5673.1.S1_a_at	5.021685632	POPTR_0001s38100	AT1G30500	nuclear factor Y, subunit A7 (NF-YA7); FUNCTIONS IN: sequence-specific DNA binding transcription factor activity, specific transcriptional repressor activity; INVOLVED IN: negative regulation of gene-specific transcription, regula
PtpAffx.161413.1.S1_a	4.994552872	POPTR_0003s08210	AT2G44730	Alcohol dehydrogenase transcription factor Myb/SANT-like family protein; FUNCTIONS IN: sequence-specific DNA binding transcription factor activity; INVOLVED IN: regulation of transcription; LOCATED IN: chloroplast; EXPR
PtpAffx.204675.1.S1_a	4.836189625	POPTR_0009s10400	AT4G34000	Encodes an ABA-responsive element-binding protein with similarity to transcription factors that is expressed in response to stress and abscisic acid.
PtpAffx.215479.1.S1_a	4.742175014	POPTR_0002s21570	AT4G22140	EARLY BOLTING IN SHORT DAYS (EBS); FUNCTIONS IN: DNA binding, zinc ion binding; INVOLVED IN: positive regulation of flower development, regulation of transcription, DNA-dependent, seed germination; LOCATED I
PtpAffx.204541.1.S1_s	4.612148167	POPTR_0009s13460	AT2G17870	Encodes COLD SHOCK DOMAIN PROTEIN 3 (CSP3), involved in the acquisition of freezing tolerance.
PtpAffx.101182.1.A1_a	4.609892564	POPTR_0018s03670	AT4G31420	Zinc finger protein 622; FUNCTIONS IN: sequence-specific DNA binding transcription factor activity; INVOLVED IN: regulation of transcription; LOCATED IN: intracellular; EXPRESSED IN: 25 plant structures; EXPRESSED DUF
PtpAffx.66318.2.S1_at	4.588343519	POPTR_0005s27930	AT1G42990	bZIP60 consists of a bZIP DNA binding domain followed by a putative transmembrane domain. bZIP60 mRNA is upregulated by the addition of ER stress inducers, tunicamycin (inhibitor of N-linked glycosylation), DTT (inhibitor of di
PtpAffx.203774.1.S1_a	4.446176745	POPTR_0004s03820	AT1G61110	NAC domain containing protein 25 (NAC025); FUNCTIONS IN: sequence-specific DNA binding transcription factor activity; INVOLVED IN: multicellular organismal development, regulation of transcription; LOCATED IN: cellular_
PtpAffx.153194.1.A1_a	4.417033979	POPTR_0008s06310	AT3G55730	putative transcription factor MYB109 (MYB109) mRNA,
PtpAffx.51120.1.S1_at	4.400912579	POPTR_0308s00200	AT2G20280	Zinc finger C-x8-C-x5-C-x3-H type family protein; FUNCTIONS IN: zinc ion binding, nucleic acid binding; INVOLVED IN: biological_process unknown; LOCATED IN: cellular_component unknown; EXPRESSED IN: 22 plant struc
PtpAffx.145393.2.A1_a	4.381122108	POPTR_0008s10010	AT1G48040	Protein phosphatase 2C family protein; FUNCTIONS IN: protein serine/threonine phosphatase activity, catalytic activity; INVOLVED IN: protein amino acid dephosphorylation; LOCATED IN: protein serine/threonine phosphatase com
PtpAffx.6190.1.S1_a_at	4.363794913	POPTR_0006s14160	AT1G19180	JAZ1 is a nuclear-localized protein involved in jasmonate signaling. JAZ1 transcript levels rise in response to a jasmonate stimulus. JAZ1 can interact with the COI1 F-box subunit of an SCF E3 ubiquitin ligase in a yeast-two-hybrid assa
PtpAffx.223571.1.S1_s	4.306853904	POPTR_0011s05130	AT5G16820	Encodes a putative transcription factor whose expression is not induced by heat but whose stable overexpression leads to expression of HSP. Required early in the stress response for transient expression of heat shock genes.
PtpAffx.8326.4.S1_at	4.184239416	POPTR_0008s13290	AT3G17860	JAZs are direct targets of the SCFCOI1 E3 ubiquitin-ligase and JA treatment induces their proteasome-mediated degradation. Furthermore, JAI3 negatively regulates the key transcriptional activator of JA responses, AtMYC2. The C-te
PtpAffx.92404.1.S1_at	4.169413538	POPTR_0001s27300	AT5G12840	Encodes a subunit of CCAAT-binding complex, binds to CCAAT box motif present in some plant promoter sequences. One of three members of this class (HAP2A, HAP2B, HAP2C), it is expressed in vegetative and reproductive tissue
Ptp.5425.1.S1_s_at	4.033016033	POPTR_0009s09520	AT2G14880	SWIB/MDM2 domain superfamily protein; FUNCTIONS IN: molecular_function unknown; INVOLVED IN: biological_process unknown; LOCATED IN: chloroplast; EXPRESSED IN: 22 plant structures; EXPRESSED DURING: 13
Ptp.951.1.A1_at	4.028443838	POPTR_0001s07240	AT5G07900	Mitochondrial transcription termination factor family protein; FUNCTIONS IN: molecular_function unknown; INVOLVED IN: biological_process unknown; LOCATED IN: membrane; EXPRESSED IN: 17 plant structures; EXPRESS
PtpAffx.150249.1.S1_s	0.248232244	POPTR_0001s33470	AT3G27010	Belongs to a TCP protein transcription factor family. Members of this family contain a predicted basic-helix-loop-helix domain involved in DNA binding. Related to rice PCF1 and PCF2 genes. Binds to the GCCCR element of CYCB1;
PtpAffx.203.3.A1_at	0.247068994	POPTR_0004s08480	AT5G16600	Encodes a putative transcription factor (MYB43).
PtpAffx.221077.1.S1_a	0.246872181	POPTR_0014s05010	AT2G44730	Alcohol dehydrogenase transcription factor Myb/SANT-like family protein; FUNCTIONS IN: sequence-specific DNA binding transcription factor activity; INVOLVED IN: regulation of transcription; LOCATED IN: chloroplast; EXPR
Ptp.3143.2.S1_a_at	0.244888732	POPTR_0006s10950	AT3G56400	member of WRKY Transcription Factor; Group III. Function as activator of SA-dependent defense genes and a repressor of JA-regulated genes. WRKY70-controlled suppression of JA-signaling is partly executed by NPR1.
PtpAffx.153090.1.S1_a	0.242126837	POPTR_0014s16430	AT3G07740	encodes a transcriptional adaptor ADA2a that interacts with histone acetyltransferase GCN5 homolog and CBF1
PtpAffx.120741.1.A1_a	0.241989587	POPTR_0004s04630	AT2G33810	Encodes a member of the SPL (squamosa-promoter binding protein-like)gene family, a novel gene family encoding DNA binding proteins and putative transcription factors. Contains the SBP-box, which encodes the SBP-domain, requi
PtpAffx.5175.1.A1_s_a	0.240345945	POPTR_0013s14960	AT2G38470	Member of the plant WRKY transcription factor family. Regulates the antagonistic relationship between defense pathways mediating responses to P. syringae and necrotrophic fungal pathogens. Located in nucleus. Involved in response
PtpAffx.152186.1.S1_a	0.238811513	POPTR_0001s33260	AT5G13180	Encodes a NAC domain transcription factor that interacts with VND7 and negatively regulates xylem vessel formation.
PtpAffx.141636.1.S1_a	0.237795654	POPTR_0009s02430	AT3G44350	NAC domain containing protein 61 (NAC061); CONTAINS InterPro DOMAIN/s: No apical meristem (NAM) protein (InterPro:IPR003441); BEST Arabidopsis thaliana protein match is: NAC domain containing protein 90 (TAIR:AT5
PtpAffx.54256.2.A1_s_	0.237618475	POPTR_0002s12480	AT4G24240	Encodes a Ca-dependent calmodulin binding protein. Sequence similarity to the WRKY transcription factor gene family.
PtpAffx.202730.1.S1_s	0.236730374	POPTR_0002s25920	AT5G43990	Encodes SUVR2, one of the four closely related Arabidopsis SUVR proteins that belong to the SU(VAR)3-9 subgroup of SET-domain proteins. Proteins containing the evolutionarily conserved SET domain are involved in regulation of
PtpAffx.4303.1.A1_at	0.236361416	POPTR_0010s16760	AT1G69310	member of WRKY Transcription Factor; Group II-c
Ptp.4800.1.S1_at	0.234226592	POPTR_0013s03870	AT3G04730	early auxin-induced (IAA16)
PtpAffx.139530.2.S1_s	0.233563141	POPTR_0002s00980	AT4G35550	Encodes a WUSCHEL-related homeobox gene family member with 65 amino acids in its homeodomain. WOX13 is the only family member that does not contain a sequence of eight residues (TLPLFPMH) downstream of the homeodon
PtpAffx.4542.3.S1_a_at	0.231439233	POPTR_0014s02810	AT1G45249	Leucine zipper transcription factor that binds to the abscisic acid (ABA)?responsive element (ABRE) motif in the promoter region of ABA-inducible genes. Enhances drought tolerance in vegetative tissues. Required for normal glucose r
PtpAffx.20290.1.S1_s_	0.231405958	POPTR_0005s21720	AT1G76710	SET domain group 26 (SDG26); FUNCTIONS IN: histone-lysine N-methyltransferase activity; INVOLVED IN: vegetative to reproductive phase transition of meristem, histone methylation; LOCATED IN: nucleus; EXPRESSED IN: 2
PtpAffx.208975.1.S1_s	0.228257619	POPTR_0010s13980	AT1G25580	Encodes suppressor of gamma response 1 (SOG1), a putative transcription factor governing multiple responses to DNA damage.
PtpAffx.203510.1.S1_a	0.227718544	POPTR_0003s22290	AT1G51140	Encodes a basic helix?loop?helix-type transcription factor involved in photoperiodism flowering.
Ptp.3665.1.S1_s_at	0.227079383	POPTR_0016s01980	AT3G22170	A component of the PHYA signaling network, mediates the FR-HIR response to far-red light in concert with FAR1.
PtpAffx.160182.1.A1_a	0.22503063	POPTR_0016s00940	AT4G16420	Transcriptional co-activator. Essential for the developmental switch from cell proliferation to cell differentiation in response to variations in auxin and cytokinin concentrations.
PtpAffx.201437.1.S1_a	0.224468553	POPTR_0001s46070	AT2G17410	ARID/BRIGHT DNA-binding domain-containing protein; FUNCTIONS IN: sequence-specific DNA binding transcription factor activity; INVOLVED IN: regulation of transcription; LOCATED IN: intracellular; EXPRESSED IN: 25 p
Ptp.5973.1.S1_s_at	0.222564776	POPTR_0004s04720	AT5G44210	encodes a member of the ERF (ethylene response factor) subfamily B-1 of ERF/AP2 transcription factor family (ATERF-9). The protein contains one AP2 domain. There are 15 members in this subfamily including ATERF-3, ATERF-4
PtpAffx.7050.1.S1_a_at	0.222125759	POPTR_0001s24840	AT1G07540	Arabidopsis thaliana telomere-binding protein, putative (At1g07540)
PtpAffx.4624.1.S1_at	0.221677089	POPTR_0008s23260	AT3G16770	Encodes a member of the ERF (ethylene response factor) subfamily B-2 of the plant specific ERF/AP2 transcription factor family (RAP2.3). The protein contains one AP2 domain. There are 5 members in this subfamily including RAP2
Ptp.5104.1.S1_s_at	0.221416668	POPTR_0005s20240	AT1G01720	Belongs to a large family of putative transcriptional activators with NAC domain. Transcript level increases in response to wounding and abscisic acid. ATAF1 attenuates ABA signaling and sythesis. Mutants are hyposensitive to ABA.
PtpAffx.31202.1.S1_at	0.220755758	POPTR_0018s12200	AT5G25220	A member of class II knotted1-like homeobox gene family (together with KNAT4 and KNAT5). Expressed in: hypocotyl-root boundary, anther-filament junction in flowers, ovule-funiculus and peduncle-silique boundaries, petioles and
PtpAffx.39778.1.A1_at	0.22000237	POPTR_0008s16110	AT4G14550	IAA14 is a member of the Aux/IAA protein family. Involved in lateral root development. Gain of function mutation decreases auxin-inducible gene expression. Protein is localized to the nucleus. Expressed in stele and root tip epidermi
PtpAffx.19179.2.A1_a_	0.218590915	POPTR_0007s14790	AT3G49800	BSD domain-containing protein; FUNCTIONS IN: molecular_function unknown; INVOLVED IN: biological_process unknown; LOCATED IN: cellular_component unknown; EXPRESSED IN: cultured cell; CONTAINS InterPro DOM
PtpAffx.1627.1.S1_a_at	0.218514652	POPTR_0002s05630	AT2G43010	Isolated as a semidominant mutation defective in red -light responses. Encodes a nuclear localized bHLH protein that interacts with active PhyB protein. Negatively regulates phyB mediated red light responses. Involved in shade avoida
PtpAffx.18604.1.S1_at	0.217346549	POPTR_0012s03530	AT1G18330	EARLY-PHYTOCHROME-RESPONSIVE1
PtpAffx.107323.1.S1_s	0.214232376	POPTR_0014s04080	AT1G65910	NAC domain containing protein 28 (NAC028); FUNCTIONS IN: sequence-specific DNA binding transcription factor activity; INVOLVED IN: multicellular organismal development, regulation of transcription; LOCATED IN: cellular_
PtpAffx.205522.1.S1_x	0.213574615	POPTR_0005s15990	AT4G36020	Encodes a cold shock domain protein. Involved in cold acclimation by blocking the secondary structure of mRNA which in turn facilitates translation at cold temperature.
PtpAffx.200993.1.S1_s	0.211348358	POPTR_0001s35980	AT5G14370	CCT motif family protein; LOCATED IN: chloroplast; EXPRESSED IN: 21 plant structures; EXPRESSED DURING: 13 growth stages; CONTAINS InterPro DOMAIN/s: CCT domain (InterPro:IPR010402); BEST Arabidopsis thalian
Ptp.4550.1.S1_at	0.210899043	POPTR_0014s10260	AT1G01060	LHY encodes a myb-related putative transcription factor involved in circadian rhythm along with another myb transcription factor CCA1
PtpAffx.86833.1.A1_at	0.208900341	POPTR_0009s01020	AT2G28200	C2H2-type zinc finger family protein; FUNCTIONS IN: sequence-specific DNA binding transcription factor activity, zinc ion binding, nucleic acid binding; INVOLVED IN: regulation of transcription; LOCATED IN: intracellular; EXP
Ptp.1550.1.S1_at	0.203693291	POPTR_0010s08880	AT3G23050	Transcription regulator acting as repressor of auxin-inducible gene expression. Plays role in the control of gravitropic growth and development in light-grown seedlings. Auxin induces the degradation of the protein in a dosage-dependent
Ptp.127.1.S1_s_at	0.202413645	POPTR_0013s03860	AT5G43700	Auxin inducible protein similar to transcription factors.
PtpAffx.7696.1.S1_at	0.202100194	POPTR_0010s08890	AT5G43700	Auxin inducible protein similar to transcription factors.
PtpAffx.18334.1.A1_at	0.202033546	POPTR_0001s01830	AT1G32130	The C-terminal portion of this protein has high homology to the C-termini of the IWS1 (Interacts With Spt6) proteins found in yeast and humans. Interacts with transcription factor BES1. Involved in brassinosteroid-regulated gene expres
PtpAffx.38516.1.A1_at	0.201954026	POPTR_0001s37620	AT4G14540	nuclear factor Y, subunit B3 (NF-YB3); FUNCTIONS IN: sequence-specific DNA binding transcription factor activity; INVOLVED IN: regulation of transcription, DNA-dependent; LOCATED IN: nucleus, intracellular; EXPRESSED
PtpAffx.37663.1.S1_at	0.201578173	POPTR_0019s04690	AT3G04070	NAC domain containing protein 47 (NAC047); FUNCTIONS IN: sequence-specific DNA binding transcription factor activity; INVOLVED IN: multicellular organismal development, regulation of transcription; LOCATED IN: cellular_
PtpAffx.20746.1.S1_at	0.198273671	POPTR_0014s13780	AT5G45710	member of Heat Stress Transcription Factor (Hsf) family
PtpAffx.26834.1.S1_at	0.196810776	POPTR_0002s04620	AT4G02590	unfertilized embryo sac 12 (UNE12); FUNCTIONS IN: DNA binding, sequence-specific DNA binding transcription factor activity; INVOLVED IN: double fertilization forming a zygote and endosperm, regulation of transcription; LOC
PtpAffx.111515.1.S1_a	0.195187112	POPTR_0010s10340	AT1G69780	Encodes a homeodomain leucine zipper class I (HD-Zip I) protein.
PtpAffx.116348.1.S1_s	0.194029973	POPTR_0015s12710	AT3G02680	DNA repair and meiotic recombination protein, component of MRE11 complex with RAD50 and MRE11
PtpAffx.204146.1.S1_a	0.191371322	POPTR_0004s12290	AT3G28730	encodes a component of the FAcilitates Chromatin Transcription (FACT) complex, SSRP1. Along with STP16 binds to the promoter of FLC.
PtpAffx.14587.1.A1_at	0.184999045	POPTR_0006s10820	AT2G30400	ovate family protein 2 (OFP2); INVOLVED IN: N-terminal protein myristoylation; EXPRESSED IN: 13 plant structures; EXPRESSED DURING: 6 growth stages; CONTAINS InterPro DOMAIN/s: Protein of unknown function DUF6
PtpAffx.37625.1.A1_at	0.184848363	POPTR_0014s06190	AT4G00050	unfertilized embryo sac 10 (UNE10); FUNCTIONS IN: DNA binding, sequence-specific DNA binding transcription factor activity; INVOLVED IN: double fertilization forming a zygote and endosperm, regulation of transcription; LOC
PtpAffx.207326.1.S1_s	0.182548538	POPTR_0007s12170	AT2G23760	Encodes a member of the BEL family of homeodomain proteins. Plants doubly mutant for saw1/saw2 (blh2/blh4) have serrated leaves. BP is expressed in the serrated leaves, therefore saw2 and saw1 may act redundantly to repress BP in
PtpAffx.103616.1.S1_a	0.1816756	POPTR_0013s11510	AT1G35560	TCP family transcription factor ; FUNCTIONS IN: sequence-specific DNA binding transcription factor activity; INVOLVED IN: regulation of transcription; LOCATED IN: chloroplast; EXPRESSED IN: 16 plant structures; EXPRESSI
PtpAffx.151377.1.S1_a	0.178379548	POPTR_0005s19550	AT3G21175	member of a novel family of plant-specific GATA-type transcription factors.
PtpAffx.54126.1.S1_s_	0.177071247	POPTR_0003s16490	AT5G13180	Encodes a NAC domain transcription factor that interacts with VND7 and negatively regulates xylem vessel formation.
Ptp.620.1.S1_at	0.174463906	POPTR_0001s06870	AT3G12280	Encodes a retinoblastoma homologue RETINOBLASTOMA-RELATED protein (RBR or RBR1). RBR controls nuclear proliferation in the female gametophyte. Also required for correct differentiation of male gametophytic cell types.
PtpAffx.151555.1.A1_a	0.17221608	POPTR_0013s06390	AT1G08810	putative transcription factor of the R2R3-MYB gene family. Transcript increases under conditions that promote stomatal opening (white and blue light, abi1-1 mutation) and decreases under conditions that trigger stomatal closure (ABA,
PtpAffx.45488.2.S1_at	0.171904067	POPTR_0005s14540	AT2G01570	Member of the VHIID/DELLA regulatory family. Contains homopolymeric serine and threonine residues, a putative nuclear localization signal, leucine heptad repeats, and an LXXLL motif. Putative transcriptional regulator repressing t
PtpAffx.221984.1.S1_a	0.171608262	POPTR_0005s12130	AT1G75390	basic leucine-zipper 44 (bZIP44); FUNCTIONS IN: DNA binding, protein heterodimerization activity, sequence-specific DNA binding transcription factor activity; INVOLVED IN: regulation of transcription, DNA-dependent; LOCATI
PtpAffx.224535.1.S1_s	0.170950552	POPTR_0007s00210	AT2G44150	Encodes a protein-lysine N-methyltransferase. Located in ER.
Ptp.2409.1.S1_at	0.170608743	POPTR_0013s07550	AT5G16680	RING/FYVE/PHD zinc finger superfamily protein; FUNCTIONS IN: DNA binding, zinc ion binding; INVOLVED IN: regulation of transcription, DNA-dependent; EXPRESSED IN: 25 plant structures; EXPRESSED DURING: 13 gro
PtpAffx.201879.1.S1_a	0.167970977	POPTR_0002s08740	AT4G36020	Encodes a cold shock domain protein. Involved in cold acclimation by blocking the secondary structure of mRNA which in turn facilitates translation at cold temperature.
PtpAffx.5545.3.S1_at	0.166763764	POPTR_0002s10880	AT5G65670	auxin (indole-3-acetic acid) induced gene
PtpAffx.7696.4.S1_at	0.165456368	POPTR_0008s16100	AT5G43700	Auxin inducible protein similar to transcription factors.
Ptp.1108.1.A1_a_at	0.164155113	POPTR_0015s06480	AT1G25440	B-box type zinc finger protein with CCT domain; FUNCTIONS IN: sequence-specific DNA binding transcription factor activity, zinc ion binding; INVOLVED IN: regulation of transcription; LOCATED IN: membrane; EXPRESSED I
PtpAffx.201891.1.S1_a	0.160360633	POPTR_0002s09050	AT1G77850	Posttranscriptionally regulated by miR160 and is essential for proper development.Regulates early auxin response genes.
PtpAffx.3096.2.S1_a_at	0.159924553	POPTR_0018s04720	AT4G29190	Zinc finger C-x8-C-x5-C-x3-H type family protein; CONTAINS InterPro DOMAIN/s: Zinc finger, CCCH-type (InterPro:IPR000571); BEST Arabidopsis thaliana protein match is: CCCH-type zinc finger family protein (TAIR:AT2G19
PtpAffx.153729.1.A1_a	0.159147561	POPTR_0014s10750	AT4G01720	member of WRKY Transcription Factor; Group II-b
PtpAffx.21945.1.A1_at	0.158306733	POPTR_0002s23080	AT1G05230	Encodes a homeobox-leucine zipper family protein belonging to the HD-ZIP IV family. Mutants have trichomes that appear glass-like under a dissecting microscope as compared to the wild-type trichomes. The mutations do not affect t
PtpAffx.16432.1.S1_at	0.158263285	POPTR_0003s17260	AT3G24010	ING1 encodes a member of the Inhibitor of Growth family of nuclear-localized PhD domain containing homeodomain proteins. Binds to H3K4 di or trimethylated DNA.
Ptp.2430.1.A1_at	0.155800658	POPTR_0010s25370	AT3G20770	Encodes EIN3 (ethylene-insensitive3), a nuclear transcription factor that initiates downstream transcriptional cascades for ethylene responses.
PtpAffx.29932.1.S1_at	0.153474641	POPTR_0010s22230	AT5G60100	Encodes pseudo-response regulator 3 (APRR3/PRR3). PRR3 transcript levels vary in a circadian pattern with peak expression at dusk under long and short day conditions. PRR3 affects the period of the circadian clock and seedlings wit
PtpAffx.5224.1.A1_at	0.149245997	POPTR_0006s27950	AT4G31800	Pathogen-induced transcription factor. Binds W-box sequences in vitro. Forms protein complexes with itself and with WRKY40 and WRKY60. Constitutive expression of WRKY18 enhanced resistance to P. syringae, but its coexpressi
PtpAffx.205854.1.S1_a	0.146769421	POPTR_0005s26800	AT1G76110	HMG (high mobility group) box protein with ARID/BRIGHT DNA-binding domain; FUNCTIONS IN: sequence-specific DNA binding transcription factor activity; INVOLVED IN: regulation of transcription; LOCATED IN: intracellu
PtpAffx.200785.1.S1_s	0.146712538	POPTR_0001s24850	AT1G07530	Encodes a member of the GRAS family of transcription factors. The protein interacts with the TGA2 transcription factor and affects the transcription of stress-responsive genes. The protein is found in the nucleus and is also exported to t
PtpAffx.32240.1.S1_at	0.145946045	POPTR_0003s17340	AT3G24050	Encodes a member of the GATA factor family of zinc finger transcription factors.
Ptp.128.1.S1_at	0.145799744	POPTR_0005s23980	AT4G14550	IAA14 is a member of the Aux/IAA protein family. Involved in lateral root development. Gain of function mutation decreases auxin-inducible gene expression. Protein is localized to the nucleus. Expressed in stele and root tip epidermi
PtpAffx.99810.1.A1_a_	0.145454366	POPTR_0006s13530	AT3G09600	Encodes a MYB-like transcription factor similar to CIRCADIAN CLOCK-ASSOCIATED1 (CCA1) and ELONGATED HYPOCOTYL (LHY). Involved in the regulation of circadian clock by modulating the pattern of histone 3 (H3) ac
PtpAffx.7785.1.A1_at	0.145365907	POPTR_0006s28350	AT5G24930	CONSTANS-like 4 (COL4); FUNCTIONS IN: sequence-specific DNA binding transcription factor activity, zinc ion binding; INVOLVED IN: regulation of transcription; LOCATED IN: intracellular, chloroplast; EXPRESSED IN: 21 p
Ptp.1032.1.S1_at	0.145329145	POPTR_0004s10800	AT3G02380	homologous to the flowering-time gene CONSTANS (CO) encoding zinc-finger proteins
PtpAffx.15802.1.S1_at	0.143440558	POPTR_0007s13700	AT5G66320	Encodes GATA transcription factor gene GNC, involved in regulating carbon and nitrogen metabolism. Expression occurs in aerial tissue at an early stage of development and is inducible by nitrate.
PtpAffx.10843.2.A1_s_	0.14275541	POPTR_0010s13490	AT1G68520	B-box type zinc finger protein with CCT domain; FUNCTIONS IN: sequence-specific DNA binding transcription factor activity, zinc ion binding; INVOLVED IN: regulation of transcription; LOCATED IN: intracellular; EXPRESSED
Ptp.3323.1.S1_s_at	0.140463097	POPTR_0003s15040	AT5G51190	encodes a member of the ERF (ethylene response factor) subfamily B-3 of ERF/AP2 transcription factor family. The protein contains one AP2 domain. There are 18 members in this subfamily including ATERF-1, ATERF-2, AND ATE
PtpAffx.158042.2.A1_a	0.135666814	POPTR_0015s00860	AT5G53660	Growth regulating factor encoding transcription activator. One of the nine members of a GRF gene family, containing nuclear targeting domain. Involved in leaf development and expressed in shoot and flower.
PtpAffx.200918.1.S1_s	0.133031702	POPTR_0001s28850	AT1G07050	CCT motif family protein; CONTAINS InterPro DOMAIN/s: CCT domain (InterPro:IPR010402); BEST Arabidopsis thaliana protein match is: CCT motif family protein (TAIR:AT4G25990.1); Has 1772 Blast hits to 1772 proteins in 1
PtpAffx.2179.1.S1_at	0.130573166	POPTR_0007s04780	AT1G01720	Belongs to a large family of putative transcriptional activators with NAC domain. Transcript level increases in response to wounding and abscisic acid. ATAF1 attenuates ABA signaling and sythesis. Mutants are hyposensitive to ABA.
PtpAffx.121626.1.A1_a	0.13006935	POPTR_0010s17650	AT1G69600	Encodes ZFHD1, a member of the zinc finger homeodomain transcriptional factor family. Binds to the 62 bp promoter region of ERD1 (early responsive to dehydration stress 1). Expression of ZFHD1 is induced by drought, high salinit
PtpAffx.53113.1.S1_at	0.12504142	POPTR_0009s12000	AT4G34530	Encodes a transcription factor CIB1 (cryptochrome-interacting basic-helix-loop-helix). CIB1 interacts with CRY2 (cryptochrome 2) in a blue light-specific manner in yeast and Arabidopsis cells, and it acts together with additional CIB1-
PtpAffx.10586.1.S1_at	0.119310227	POPTR_0003s18060	AT1G80840	Pathogen-induced transcription factor. Binds W-box sequences in vitro. Forms protein complexes with itself and with WRKY40 and WRKY60. Coexpression with WRKY18 or WRKY60 made plants more susceptible to both P. syringa
PtpAffx.570.1.S1_at	0.112654773	POPTR_0012s12760	AT2G40140	CZF1; CONTAINS InterPro DOMAIN/s: Zinc finger, CCCH-type (InterPro:IPR000571), Ankyrin repeat-containing domain (InterPro:IPR020683), Ankyrin repeat (InterPro:IPR002110); BEST Arabidopsis thaliana protein match is: sal
PtpAffx.159337.1.A1_a	0.11171477	POPTR_0015s11550	AT5G50915	basic helix-loop-helix (bHLH) DNA-binding superfamily protein; FUNCTIONS IN: DNA binding, sequence-specific DNA binding transcription factor activity; INVOLVED IN: response to gibberellin stimulus; LOCATED IN: nucleus;
PtpAffx.201804.1.S1_s	0.108641452	POPTR_0002s06920	AT1G76880	Duplicated homeodomain-like superfamily protein; CONTAINS InterPro DOMAIN/s: SANT, DNA-binding (InterPro:IPR001005), MYB-like (InterPro:IPR017877); BEST Arabidopsis thaliana protein match is: Duplicated homeodoma
PtpAffx.50494.1.A1_at	0.104478968	POPTR_0006s02840	AT1G73100	Encodes a SU(VAR)3-9 homolog, a SET domain protein. Known SET domain proteins are involved in epigenetic control of gene expression and act as histone methyltransferases. There are 10 SUVH genes in Arabidopsis and member
Ptp.1500.1.A1_at	0.104202045	POPTR_0009s13640	AT4G38620	Encodes a R2R3 MYB protein which is involved in the response to UV-B. It functions as a repressor of target gene expression. One of its target genes encodes cinnamate 4-hydroxylase; mutants accumulate sinapate esters in their leave
PtpAffx.62380.1.S1_s_	0.102888682	POPTR_0010s19520	AT2G40140	CZF1; CONTAINS InterPro DOMAIN/s: Zinc finger, CCCH-type (InterPro:IPR000571), Ankyrin repeat-containing domain (InterPro:IPR020683), Ankyrin repeat (InterPro:IPR002110); BEST Arabidopsis thaliana protein match is: sal
PtpAffx.24588.2.A1_a_	0.102296715	POPTR_0007s03840	AT5G09330	NAC domain containing protein 82 (NAC082); FUNCTIONS IN: sequence-specific DNA binding transcription factor activity; INVOLVED IN: multicellular organismal development, regulation of transcription; LOCATED IN: cellular_
PtpAffx.25830.1.A1_at	0.100946702	POPTR_0009s03850	AT1G07530	Encodes a member of the GRAS family of transcription factors. The protein interacts with the TGA2 transcription factor and affects the transcription of stress-responsive genes. The protein is found in the nucleus and is also exported to t

PtpAffx.89274.1.A1_at	0.096341684	POPTR_0018s02170	AT4G27910	SET domain protein 16 (SDG16); FUNCTIONS IN: zinc ion binding; EXPRESSED IN: 24 plant structures; EXPRESSED DURING: 12 growth stages; CONTAINS InterPro DOMAIN/s: SET domain (InterPro:IPR001214), Zinc finger,
Ptp.5332.1.S1_at	0.095355487	POPTR_0014s01760	AT1G27730	Related to Cys2/His2-type zinc-finger proteins found in higher plants. Compensated for a subset of calcineurin deficiency in yeast. Salt tolerance produced by ZAT10 appeared to be partially dependent on ENA1/PMR2, a P-type ATPase
PtpAffx.4817.1.S1_at	0.095046763	POPTR_0019s11870	AT1G68810	basic helix-loop-helix (bHLH) DNA-binding superfamily protein; FUNCTIONS IN: DNA binding, sequence-specific DNA binding transcription factor activity; INVOLVED IN: regulation of transcription; LOCATED IN: nucleus; EXP
PtpAffx.211077.1.S1_a	0.093996729	POPTR_0013s05670	AT3G04030	Homeodomain-like superfamily protein; FUNCTIONS IN: sequence-specific DNA binding transcription factor activity; INVOLVED IN: regulation of transcription; EXPRESSED IN: 9 plant structures; EXPRESSED DURING: 4 anthes
PtpAffx.68429.1.S1_a_	0.093084881	POPTR_0004s05920	AT1G29280	member of WRKY Transcription Factor; Group II-e
PtpAffx.938.1.A1_at	0.089021161	POPTR_0013s03800	AT5G28770	bZIP protein BZO2H3 mRNA, partial cds
PtpAffx.289.1.S1_at	0.086983345	POPTR_0017s14410	AT3G02380	homologous to the flowering-time gene CONSTANS (CO) encoding zinc-finger proteins
PtpAffx.87591.1.S1_at	0.085756363	POPTR_0010s15280	AT5G49450	Encodes a transcription activator is a positive regulator of plant tolerance to salt, osmotic and drought stresses.
PtpAffx.124024.1.S1_s	0.082583159	POPTR_0012s09990	AT3G48550	BEST Arabidopsis thaliana protein match is: C2H2-like zinc finger protein (TAIR:AT2G01940.3); Has 78 Blast hits to 78 proteins in 11 species: Archae - 0; Bacteria - 0; Metazoa - 0; Fungi - 0; Plants - 78; Viruses - 0; Other Eukaryotes
PtpAffx.2179.2.S1_at	0.076670273	POPTR_0005s07060	AT1G01720	Belongs to a large family of putative transcriptional activators with NAC domain. Transcript level increases in response to wounding and abscisic acid. ATAF1 attentuates ABA signaling and sythesis. Mutants are hyposensitive to ABA.
PtpAffx.14623.1.A1_at	0.074254247	POPTR_0007s00880	AT1G05690	BTB and TAZ domain protein. Acts redunantly with BT1 and BT2 during female gametophyte development. Acts with BT2 during male gametophyte development.
PtpAffx.147430.1.A1_a	0.073557234	POPTR_0012s13870	AT5G51990	encodes a member of the DREB subfamily A-1 of ERF/AP2 transcription factor family (CBF4). The protein contains one AP2 domain. There are six members in this subfamily, including CBF1, CBF2, and CBF3. This gene is involved i
Ptp.3894.1.S1_s_at	0.070824409	POPTR_0001s24250	AT2G37430	C2H2 and C2HC zinc fingers superfamily protein; FUNCTIONS IN: sequence-specific DNA binding transcription factor activity, zinc ion binding, nucleic acid binding; INVOLVED IN: response to chitin, regulation of transcription; LC
PtpAffx.217349.1.S1_x	0.070788661	POPTR_0004s00890	AT1G62300	Encodes a transcription factor WRKY6. Regulates Phosphate1 (Pho1) expression in response to low phosphate (Pi) stress.
PtpAffx.68429.2.S1_at	0.068507647	POPTR_0011s06990	AT1G29280	member of WRKY Transcription Factor; Group II-e
PtpAffx.81893.1.A1_at	0.063256074	POPTR_0005s24460	AT3G23240	encodes a member of the ERF (ethylene response factor) subfamily B-3 of ERF/AP2 transcription factor family (ERF1). The protein contains one AP2 domain. There are 18 members in this subfamily including ATERF-1, ATERF-2, AN
PtpAffx.159345.1.A1_a	0.059324839	POPTR_0006s23480	AT1G19210	encodes a member of the DREB subfamily A-5 of ERF/AP2 transcription factor family. The protein contains one AP2 domain. There are 15 members in this subfamily including RAP2.1, RAP2.9 and RAP2.10.
PtpAffx.37783.1.A1_s_	0.057876365	POPTR_0016s14490	AT3G56400	member of WRKY Transcription Factor; Group III. Function as activator of SA-dependent defense genes and a repressor of JA-regulated genes. WRKY70-controlled suppression of JA-signaling is partly executed by NPR1.
PtpAffx.135358.1.S1_s	0.049511417	POPTR_0001s11780	AT5G51190	encodes a member of the ERF (ethylene response factor) subfamily B-3 of ERF/AP2 transcription factor family. The protein contains one AP2 domain. There are 18 members in this subfamily including ATERF-1, ATERF-2, AND ATE
PtpAffx.203341.1.S1_s	0.032036091	POPTR_0003s15030	AT4G17500	Encodes a member of the ERF (ethylene response factor) subfamily B-3 of ERF/AP2 transcription factor family (ATERF-1). The protein contains one AP2 domain. There are 18 members in this subfamily including ATERF-1, ATERF-2
PtpAffx.42822.1.A1_at	0.029558039	POPTR_0001s11800	AT5G07580	encodes a member of the ERF (ethylene response factor) subfamily B-3 of ERF/AP2 transcription factor family. The protein contains one AP2 domain. There are 18 members in this subfamily including ATERF-1, ATERF-2, AND ATE
PtpAffx.572.3.S1_a_at	0.015210678	POPTR_0010s13930	AT1G25560	Encodes a member of the RAV transcription factor family that contains AP2 and B3 binding domains. Involved in the regulation of flowering under long days. Loss of function results in early flowering. Overexpression causes late flowe

to pathogen infe
to drought.

to drought.
nably by stimulating
IN: male gametophyte, pollen tube; EXI

SED DURING: 13 growth stages; CONTAINS Int
SSED DURING: 15 growth stages; CONTAINS In
I: nucleus; EXPRESSED IN: 22 plant
tor, Myb-type, DNA-binding (InterPro:IPR0179

inus and part o
nplex; EXPRESSED IN: 23 plant st

essed abundantly d
3: 13 growth stages; CONTAINS InterPro DOMAIN/s: Ti
Blast hits to 1364 proteins in 12
r protein (TAIR:AT5G52010.1); Ha
OCATED IN: nucleus; CONTAINS Int
50.1); Has 635 Blast hits to 634 proteins
iterPro:IPR001878), HTH transcriptional regul
ase of decrease
SSED DURING: 15 growth stages; CONTAINS In
r factor Y, subunit C1 (TAIR:AT3G
otein (TAIR:AT2G38250.1); Has 1807 Blast h
ayers. Loss of function

ation of transcription, DN
ESSED IN: 22 plant structures; EXPRI

N: cellular_component unknown; EXPRESSED IN: 2

RING: 15 growth stages; CONTAINS Inter
isulfide bond formation) and
_component unknown; EXPRESSED IN: 6 p

ctures; EXPRESSED DURING: 13 growth stage
nplex; EXPRESSED IN: 23 plant st
ay only in the prese

rminal portion of JAZ3,
s.
growth stages; CONTAINS InterPro DOMAIN/s: SWIB/MDM
ED DURING: 11 growth stages; CONTAINS InterP
l. Involved in regulation of e

ESSED IN: 22 plant structures; EXPRI

ed and sufficient for interac
to various abiotic stre

G22380.1); Has 30201 Blast hits to 17322 p

eukaryotic gene expression a

nain called the WUS box.
response. Localized in t
3 plant structures; EXPRESSED DURING: 1

lant structures; EXPRESSED DURING: 13 grov
, ATERF-7, and leafy petiole

2.2 AND RAP2.12.It is localiz

root. Light-regulated expres
s. Functions as a ne
MAIN/s: BSD (InterPro:IPR005607); BEST Arabidopsis
nce response. Protein

_component unknown; EXPRESSED IN: 19

a protein match is: CCT motif family protein (TA

PRESSED IN: 20 plant structures
t manner in a proces

ssion.
IN: 21 plant structures; EXPRESSED DUF
_component unknown; EXPRESSED IN: 16

ATED IN: nucleus; EXPRESSED IN: 2

23 (InterPro:IPR006458); BEST Arabidopsis tha
ATED IN: nucleus; EXPRESSED IN: 2
leaves.
ED DURING: 11 growth stages; CONTAINS

Regulates stem cell maintenance in
desiccation, darkness
he gibberellin response a
ED IN: chloroplast; CONTAINS Inte

wth stages; CONTAINS InterPro DOMAIN/s: Zinc

N: leaf; CONTAINS InterPro DOMAIN/s: C

810.1); Has 726 Blast hits to 700 prot

richome growth or branch

reduced levels of PRR3
on with WRKY40 or WRKY60 made
lar, nucleus; EXPRESSED IN: 21 plant str
he cytoplasm.

s. Functions as a ne
etylation
plant structures; EXPRESSED DURING: 13

IN: 21 plant structures; EXPRESSED
RF-5

05 species: Archae - 0; Bacteria - 0;

ty and abscisic acid.
-related proteins to
e and B. cinerea. WRKY18, WRKY40, and
t-inducible zinc finger 1 (TAIR:AT3G
; EXPRESSED IN: 23 plant structures;
in-like superfamily protein (TAIR:AT1G7689
s of this subfamily of the SET pr
s. MYB4 binds to its o
t-inducible zinc finger 1 (TAIR:AT3G
_component unknown; EXPRESSED IN: 24
he cytoplasm.

PHD-type, conserved site (InterPro:IPR019786)
required for Li+ and Na+
RESSED IN: 20 plant structures; EXPRES
sis, 4 leaf senescence stage, petal diffe

- 0 (source: NCBI BI

n response to drought stress and
OCATED IN: intracellular; EXP

ND ATERF-5. EREBP like protein tha

RF-5
, AND ATERF-5
RF-5
ring and repression of
