# Supplementary material for: Effects of high temperature on photosynthesis and related gene expression in poplar
Source: BMC Plant Biol. 2014 Apr 28;14:111. doi: 10.1186/1471-2229-14-111 (PMC4036403; doi:10.1186/1471-2229-14-111)
Supplement: Additional file 6 — Differentially expressed HSF and HSP genes. [file 1471-2229-14-111-S6.pdf]

Additional file 6 Candidate genes involved in hormone metabolism

Probe	Foldchange	Poplar Gene model	TAIR gene model		Annotation
Ptp.4828.1.S1_at	2.502686342	POPTR_0007s10980	AT5G67030	ABA1	Encodes a single copy zeaxanthin epoxidase gene that functions in first step of the biosynthesis of the abiotic stress hormon...
PtpAffx.63251.1.A1_at	0.484244292	POPTR_0001s06170	AT1G52340	ABA2	Encodes a cytosolic short-chain dehydrogenase/reductase involved in the conversion of xanthoxin to ABA-aldehyde during A
PtpAffx.86629.1.S1_at	0.91685893	POPTR_0007s08330	AT1G16540	ABA3	Encodes molybdenum cofactor sulfurase. Involved in Moco biosynthesis. Involved in the conversion of ABA-aldehyde to AB
Ptp.5483.2.S1_a_at	0.4540666	POPTR_0004s09860	AT1G67080	ABA4	Encodes a protein involved in the photoprotection of PSII. An aba4-1 mutant completely lacks neoxanthin,a component of the
PtpAffx.209758.1.S1_at	0.388959624	POPTR_0011s02230	AT4G18350	NCED2	Encodes 9-cis-epoxycarotenoid dioxygenase, a key enzyme in the biosynthesis of abscisic acid. The expression of this g....
PtpAffx.216536.1.S1_at	3.628708253	POPTR_0001s40420	AT3G14440	NCED3	Encodes 9-cis-epoxycarotenoid dioxygenase, a key enzyme in the biosynthesis of abscisic acid. Regulated in response t....
PtpAffx.101087.1.S1_at	0.290477374	Potri.005G069100	AT4G19170	NCED4	chloroplast-targeted member of a family of enzymes similar to nine-cis-epoxycarotenoid dioxygenase
PtpAffx.222439.1.S1_at	1.478146749	POPTR_0004s20280	AT2G27150	AAO3	Encodes the aldehyde oxidase delta isoform catalyzing the final step in abscisic acid biosynthesis.
PtpAffx.204443.1.S1_s_at	0.055795385	POPTR_0016s14100	AT5G20960	AAO1	calcineurin B-like-interacting protein
PtpAffx.204529.1.S1_at	1.382233039	POPTR_0009s13680	AT1G52400	BG1	encodes a member of glycosyl hydrolase family 1, located in inducible ER bodies which were formed after wounding, require
Ptp.1674.1.A1_s_at	0.058326485	POPTR_0019s11260	AT1G71960	ABCG25	Encodes a plasma membrane localized ABC transporter involved in abscisic acid transport and responses.
Ptp.2310.1.A1_s_at	0.887260234	POPTR_0001s14650	AT1G15520	ABCG40	ABC transporter family involved in ABA transport and resistance to lead. Localizes to plasma membrane. Upregulated by lea
PtpAffx.112212.1.S1_at	0.174241459	POPTR_0002s12770		CYP707A14	CYTOCHROME P450, FAMILY 707, SUBFAMILY A, POLYPEPTIDE 14
PtpAffx.155137.1.S1_at	5.008958677	POPTR_0004s14820		CYP707A5	CYTOCHROME P450, FAMILY 707, SUBFAMILY A, POLYPEPTIDE 5
PtpAffx.200780.1.S1_s_at	8.931353764	POPTR_0004s24360		CYP707A6	CYTOCHROME P450, FAMILY 707, SUBFAMILY A, POLYPEPTIDE 6
PtpAffx.74008.1.S1_s_at	2.059037675	Potri.009G049500		CYP707A12v1	CYTOCHROME P450, FAMILY 707, SUBFAMILY A, POLYPEPTIDE 12
PtpAffx.94716.1.A1_at	0.052227763	POPTR_0005s03580	AT1G55020	LOX1	lipoxygenase, a defense gene conferring resistance Xanthomonas campestris
Ptp.5224.1.S1_x_at	31.4477382	POPTR_0004s10240	AT3G25780	AOC3	Encodes allene oxide cyclase, one of the enzymes involved in jasmonic acid biosynthesis. One of four genes in Arabidopsis t

BA bi...
A, the ...
ch...

d in...

hat encode this enzyme. mRNA expression is upregulated in senescing leaves. Note: Nomenclature for Arabidopsis allene oxide cy
